# Data Mining Sheds Light on a Novel Family of Plant-Associated Negative-Sense RNA Viruses Linked to Lispiviruses

**DOI:** 10.3390/v18101113

**Published:** 2026-10-08

**Authors:** Nicolás Bejerman, David Huebert, Robert Alvarez-Quinto, Diego Quito-Avila, Humberto Debat

**Affiliations:** 1 Instituto de Patología Vegetal, Centro de Investigaciones Agropecuarias, Instituto Nacional de Tecnología Agropecuaria (IPAVE-CIAP-INTA), Cordoba 5020, Argentina; 2 Consejo Nacional de Investigaciones Científicas y Técnicas, Unidad de Fitopatología y Modelización Agrícola, Cordoba 5020, Argentina; 3 Department of Molecular Genetics, University of Toronto, Toronto, ON M5S 3E2, Canada; 4 Department of Plant Pathology, University of Minnesota, Minneapolis, MN 55108, USA; 5 Facultad de Ciencias de la Vida, Escuela Superior Politécnica del Litoral, R2XV+55, Guayaquil 090902, Ecuador

**Keywords:** negative-sense, virus taxonomy, metatranscriptomics, virus discovery, lispiviruses

## Abstract

Large-scale mining of public transcriptomic datasets can reveal viral diversity that remains invisible to conventional virus-surveillance approaches, while increasingly powerful structure-prediction methods provide a complementary route to characterizing highly divergent viral proteins. Here, we combine sequence detection, phylogenetic analysis, structural prediction, and host-association analyses to investigate the cryptic diversity and biology of a new clade of plant-associated lispi-like viruses. Analyses of RNA-sequencing datasets identified and enabled assembly of 87 coding-complete lispi-like virus genome sequences associated with 75 plant hosts, expanding the known diversity of this new group by approximately 40-fold. The viruses share a conserved four-cistron genome organization, 3′-N(P1)-P2-P3-L(P4)-5′, in which the first cistron is referred to interchangeably as N or P1 and the fourth as L or P4. Structural analyses generate testable structural hypotheses for the four conserved proteins. N (P1) adopts a canonical negative-strand RNA virus nucleocapsid architecture with conserved RNA-interacting residues and a predicted RNA-packaging configuration. P2 is exceptionally divergent, although a subset of structures resembles the ITPase/HAM1 fold. P3 forms a conserved trimeric coiled-coil architecture reminiscent of a viral fusion-protein stalk, but lacks the family-wide sequence features expected of a canonical membrane glycoprotein. L (P4) contains a structurally resolved Mononegavirales-type RNA-dependent RNA polymerase (RdRp) core with invariant catalytic motifs, including the characteristic GDN signature, whereas its accessory regions are substantially more divergent. Phylogenetic insights form a distinct monophyletic lineage sister to the predominantly invertebrate-associated *Lispiviridae*, supporting their recognition as the new proposed family Masuviridae, comprising 15 tentative genera. Genus-level clustering is accompanied by marked differences in host association, ranging from strong specialization to broader host ranges. Retrospective screening of public sequencing libraries further identified lispi-like virus sequences in 1536 libraries representing 134 plant species, 11 plant families and 182 geographic locations, highlighting a substantial and geographically widespread cryptic virome. Together, these results establish Masuviridae as a deeply divergent lineage of plant-associated negative-sense RNA viruses and illustrate how the integration of sequence, structural, and large-scale transcriptomic analyses can move viral dark matter from detection towards evolutionary and functional characterization.

## 1. Introduction

In the current era of metagenomics, the rapid discovery of novel viruses has revealed a vast and diverse evolutionary landscape of replicating entities, presenting significant challenges in their systematic classification [1]. In response to this complexity, various strategies have emerged, culminating in a comprehensive proposal for establishing a “megataxonomy” of the viral world [2].

Despite extensive efforts to characterize the viral component of the biosphere, it is evident that only a tiny fraction, likely less than one percent, of viral diversity has been thoroughly explored [3,4,5,6]. As a result, our understanding of the global virome remains limited, particularly regarding its immense diversity and the interactions between viruses and their hosts [7,8,9,10]. To address this gap, researchers have increasingly turned to mining publicly available transcriptome datasets derived from High-Throughput Sequencing (HTS), which offers a rapid and cost-effective approach to viral discovery [8,11,12,13,14]. This data-driven strategy has proven particularly valuable given the sheer volume of freely available datasets within the Sequence Read Archive (SRA), maintained by the National Center for Biotechnology Information (NCBI), which continues to expand at an extraordinary pace. Although the data in SRA represent a partial and potentially biased sample of Earth’s biodiversity, the NCBI-SRA remains an exceptionally efficient and economical resource for the identification of novel viruses [15]. The tool ‘Serratus’ [8] has proven invaluable, enabling large-scale data mining and accelerating viral sequence discovery at an unprecedented pace.

In the realm of virus taxonomy, a growing consensus has emphasized the necessity of incorporating viruses identified solely through metagenomic data into the official classification framework of the International Committee on Taxonomy of Viruses (ICTV) [16]. This shift underscores the vital role of metagenomic approaches in expanding our understanding of the global virome and modernizing taxonomic systems to accommodate the ever-growing known diversity of viruses [17].

Negative-sense RNA viruses constitute a comparatively underexplored component of the plant virosphere [18]. In contrast to positive-sense RNA viruses, whose diversity has been extensively documented through both disease surveillance and metatranscriptomic surveys, plant-associated negative-sense viruses have historically been represented by a smaller number of taxonomic lineages [5,6,18] (https://ictv.global/taxonomy, accessed on 30 July 2026). Recent transcriptome-mining studies, however, have begun to reveal a broader and more complex diversity of plant-associated negative-sense viruses, including viruses detected in asymptomatic hosts and lineages that lack established taxonomic or biological frameworks [11,19,20]. This expanding diversity raises a fundamental question: to what extent does the apparent paucity of plant-associated negative-sense RNA virus lineages reflect biological rarity, and to what extent does it reflect limitations in sampling and sequence-based discovery?

The rapid expansion of viral sequence space has also exposed a second challenge: identifying a viral sequence is increasingly easier than determining what its encoded proteins do. Viral proteins often evolve rapidly, and sequence divergence can erase recognizable homology even when structural and functional constraints are retained [21,22]. This problem is particularly acute for newly discovered RNA viruses, for which many proteins have no reliable sequence-based annotation and therefore remain part of the viral functional “dark matter” [23,24]. Recent large-scale structural analyses have shown that predicted protein structures can recover deeply conserved folds and reveal relationships that are undetectable from primary sequence alone, providing a complementary route to functional and evolutionary inference [21,22,23,24]. Structure-guided approaches are therefore especially valuable for newly discovered viral lineages in which conventional homology searches provide little information, allowing conserved architectural features to be distinguished from genuinely lineage-specific proteins while also defining the limits of functional inference.

The order *Mononegavirales* was created to accommodate viruses with linear, single-stranded, negative-sense RNA genomes [25]. Currently, this order is composed of 11 families, where the only family to date to harbor plant-associated viruses is the *Rhabdoviridae* [26]. *Lispiviridae* is a family encompassing viruses with negative-sense RNA genomes of 6.5–15.5 kb which have mainly been identified in arthropods [27]. Genomes of members of the family *Lispiviridae* commonly have five to six open reading frames (ORFs). Encoded proteins likely include a nucleocapsid (N), a phosphoprotein (P), a glycoprotein (G), and a large protein (L) including an RNA-directed RNA polymerase (RdRP) domain [27]. Currently, the family *Lispiviridae* includes 30 genera with 45 species (https://ictv.global/taxonomy, accessed on 30 July 2026). In 2018, a novel rod-shaped virus with a negative-sense single-stranded genome was identified in maize samples collected from Ecuador. The virus, tentatively named maize suscal virus (MSuV), had a 12 kb genome comprising four ORFs with the predicted L protein phylogenetically related to lispiviruses [28]. Later, the L gene of a novel lispivirus-like virus associated with the plant host *Disporopsis* spp. was identified by [29], who proposed that both viruses may belong to a new sister lineage of the family *Lispiviridae*, for which we propose the name Masuviridae within the order *Mononegavirales*.

Here, we combine large-scale mining of public RNA-sequencing datasets with phylogenomic, host-association, and structure-guided analyses to characterize the diversity and biology of plant-associated lispi-like viruses. We identified and assembled 87 lispi-like virus coding sequences associated with 75 plant hosts and used comparative phylogenetic analyses to establish their evolutionary relationship with *Lispiviridae* and to develop a proposed taxonomic framework for this lineage. We then examine the predicted structures of all four major viral proteins to distinguish conserved viral architectures from lineage-specific and unresolved regions and to generate testable hypotheses about their functions. Lastly, we use large-scale retrospective screening of public RNA-sequencing libraries to assess the broader distribution, host range, and apparent prevalence of these viruses. Together, these analyses move beyond sequence-based discovery to provide an integrated view of the evolutionary, structural, and ecological diversity of a previously obscure group of plant-associated negative-sense RNA viruses.

## 2. Material and Methods

### 2.1. Identification of Lispi-like Virus Sequences from Public Plant RNA-Seq Datasets

Plant-associated lispi-like virus sequences were identified by mining publicly available transcriptomic datasets. The palmprint-indexed RdRp space of the Sequence Read Archive was searched using the palmID web service of the Serratus project [8]. The predicted complete RdRp (L) protein of maize suscal virus (MSuV; GenBank accession MZ270532, protein UWX11517.1) was submitted as the query, from which palmID extracted the 113-amino-acid palmprint (barcode u64591) spanning the catalytic core from motif A to motif C, including the DFSKW, GWEGQRQKGW and QGDNQ blocks analysed in Section 3.7. This search returned 200 related palmprints from palmDB spanning 38.9–100% amino-acid identity to the MSuV palmprint, together with 1347 SRA hits distributed across 1202 sequencing runs, 185 species-like operational taxonomic units (sOTUs), 198 labelled source organisms and 255 BioProjects. The palm-ID of every hit was retrieved and searched by BLASTP 2.17.0 against the NCBI non-redundant protein database using the NCBI web service, and an sOTU was retained only when its best-scoring hit was one of the two previously described plant-associated lispi-like viruses, maize suscal virus or Disporopsis pernyi-associated lispi-like virus. Retained sOTUs were mapped to their source SRA libraries, which were re-analysed as described in Section 2.2. Searching was iterative: as coding-complete sequences of additional plant-associated lispi-like viruses were reconstructed, their predicted RdRp proteins were submitted as further palmID queries, and the same palmID retrieval and BLASTP assignment procedure was repeated until no further sOTUs satisfying the retention criterion were recovered (Supplementary Table S1). In parallel, the NCBI Transcriptome Shotgun Assembly (TSA) database was searched using the amino acid sequences of the MSuV nucleocapsid (N) and polymerase (L) proteins as queries in tBLASTn searches against Viridiplantae (taxid:33090). TSA searches used word size 6, an E-value threshold of 10, and the BLOSUM62 scoring matrix.

### 2.2. Sequence Retrieval, Assembly and Genome Reconstruction

Raw sequence reads from SRA experiments identified through Serratus were retrieved from the corresponding NCBI BioProjects (Supplementary Table S2). Reads were quality- and adapter-trimmed using Trimmomatic v0.40 [30], using standard parameters except that the quality threshold was increased from 20 to 30. The initial ILLUMINACLIP step and sliding-window quality trimming were applied, with a required average quality of 30. Filtered reads were assembled de novo using rnaSPAdes 4.3.0 with standard parameters on the Galaxy server. Resulting transcripts were screened by local BLASTX searches against MSuV RefSeq proteins using an E-value threshold of 1 × 10^−5^. Candidate viral contigs were examined manually and extended and/or validated by iterative mapping of filtered reads from the corresponding SRA library. In each iteration, reads mapping to the candidate contig were extracted and used to extend the sequence, after which the extended sequence was used as the query for the subsequent iteration. Iterations were continued until no further extension was obtained. Final extended sequences were reassembled and polished using Geneious v8.1.9 (Biomatters Ltd., Auckland, New Zealand) with high-sensitivity alignment parameters. Sequences were retained for downstream analysis when a complete coding region could be reconstructed. The resulting dataset comprised 87 lispi-like virus coding sequences, including distinct variants associated with six viruses. Sequence completeness and coding-region organization were assessed by inspection of read-supported assemblies and predicted open reading frames.

### 2.3. Genome Annotation and Comparative Sequence Analysis

Open reading frames (ORFs) were predicted using NCBI ORFfinder 0.4.3, using genetic code 1 and a minimum ORF length of 120 nt. Predicted proteins were annotated using the InterPro 110.0 protein sequence analysis platform and the NCBI Conserved Domain Database (CDD v3.20), using an E-value threshold of 0.01. Divergent proteins were further examined using HHpred v3.3.0 and HHblits v3.3.0 as implemented in the MPI Bioinformatics Toolkit. Homologous proteins were identified using BLASTP 2.17.0 searches against the NCBI non-redundant (nr) protein database. Transmembrane topology was initially assessed using TMHMM v2.0. Signal peptides were predicted using SignalP 6.0 [31]. Where appropriate, combined signal-peptide and transmembrane-topology predictions were assessed using Phobius v1.01 [32]. Nuclear localization signals were predicted using cNLS Mapper v1. All web-based prediction tools were run using their standard parameters unless otherwise specified. Protein structures were additionally searched against publicly available structural databases using Foldseek 10 [33].

### 2.4. Analysis of Genome Organization and Gene Junctions

The genomic organization of the 87 lispi-like virus sequences was determined from the reconstructed coding regions and comparative nucleotide alignments. Gene boundaries and the relative positions of the N, P2, P3, and L ORFs were compared across all sequences. To characterize conserved transcription-associated elements, nucleotide sequences surrounding each gene junction were aligned and inspected in the mRNA-sense orientation. Conserved 3′ termination/polyadenylation signals, intergenic spacers, and downstream transcription-initiation motifs were identified by comparative analysis of all available gene junctions. Motif frequencies and sequence conservation were calculated across the 87 sequences using standard sequence-alignment and nucleotide-counting procedures.

### 2.5. Pairwise Amino Acid Sequence Identity

Pairwise amino acid sequence identities were calculated for the predicted L proteins of the lispi-like viruses identified in this study and selected related viruses available in the NCBI database. Multiple sequence alignments were generated using MAFFT v7.505 [34] with standard parameters, and pairwise identities were calculated using SDTv1.2 [35]. For comparative analyses of individual viral proteins, the corresponding amino acid sequences were aligned using MAFFT 7.526 with standard parameters. Mean, median, and range of pairwise identities were calculated from the resulting alignments.

### 2.6. Phylogenetic Analysis and Taxonomic Classification

Phylogenetic analyses were performed using the predicted L protein sequences of the 87 plant-associated lispi-like viruses together with MSuV, Disporopsis pernyi-associated lispi-like virus and selected representatives of the family *Lispiviridae*. Selected rhabdovirus L proteins were included as an outgroup. Amino acid sequences were aligned using MAFFT v7.505 with the default (--auto) strategy; alignment columns were not trimmed. The resulting alignments were analyzed using MEGA11 [36]. The best-fitting amino-acid substitution model was selected in MEGA11 using the Bayesian information criterion (BIC) implemented in its Models/Find Best DNA-Protein Model routine, which identified WAG with a discrete gamma distribution of among-site rate variation and empirical amino-acid frequencies (WAG + G + F) as the best-supported model. Maximum-likelihood phylogenetic trees were then inferred under this model using the default MEGA11 heuristic search settings (nearest-neighbour interchange, with an initial tree generated by BioNJ), treating gaps and missing data by partial deletion. Node support was estimated from 1000 bootstrap replicates, and only bootstrap values above 50% are shown on the trees. Tree topology and branch support were visualized using standard phylogenetic visualization procedures. Provisional species and genus boundaries were evaluated using the combination of phylogenetic clustering and pairwise amino acid identity of the L protein. An L-protein amino acid identity threshold of 80% was used as a provisional species-level criterion within the newly proposed genera, based on the distribution of sequence identities observed among the sampled viruses. Genus-level groups were defined by discrete monophyletic clustering in the L-protein phylogeny together with marked inter-group sequence divergence.

### 2.7. Protein Structure Prediction

Three-dimensional structures of the four major viral proteins were predicted to investigate conserved structural features that could not be reliably inferred from primary sequence similarity. AlphaFold2 [37] was used to generate predicted structures for the P1, P2, and P3 proteins of the 87 lispi-like viruses. Structural predictions were generated using standard parameters and without the use of structural templates. Prediction confidence was assessed for every model using per-residue predicted local distance difference test (pLDDT) scores and, for multimeric models, predicted alignment error (PAE). pLDDT was used to stratify interpretation throughout, following the confidence bands recommended for AlphaFold2 [37]: regions with pLDDT > 90 were treated as confidently modelled, 70–90 as well modelled, 50–70 as low confidence, and <50 as effectively undetermined. Regions falling below 50 were not interpreted as evidence of a defined three-dimensional fold, and the confidence band of every structural feature discussed in the Results is reported alongside it. For P3, oligomeric models were additionally generated using AlphaFold-Multimer v2.3.2 [38] with standard parameters. Homotrimeric models were evaluated for consistency of the predicted coiled-coil architecture and overall assembly. We note explicitly that interface-confidence metrics (ipTM and interface PAE) were not used as quantitative acceptance criteria for the P3 trimers; PAE maps were inspected only qualitatively. The P3 homotrimeric models are therefore presented as architectural hypotheses rather than as validated assemblies, and this limitation is stated again in Section 3.6. A representative P4/L protein was modeled using AlphaFold2 and compared with experimentally determined structures of Mononegavirales polymerases. The representative sequence was selected to provide a structurally informative model while avoiding redundant structural analyses of highly similar variants.

### 2.8. Structure-Based Protein Annotation

Predicted protein structures were searched against experimentally determined structures in the Protein Data Bank and predicted structures in the AlphaFold Protein Structure Database using Foldseek [33]. Searches were performed in easy-search mode using the 3Di + AA alignment mode with the default sensitivity setting, querying the PDB and the AlphaFold/UniProt50 databases. Structural hits were considered interpretable only at E ≤ 1 × 10^−3^, and an assignment was accepted only when the matched fold was architecturally consistent with the query across the aligned region rather than confined to a small structural fragment. Structural matches were evaluated according to the significance and extent of structural alignment, together with agreement between the predicted fold and known protein architecture. Structural assignments were considered putative when supported by a coherent fold-level match but were not interpreted as definitive functional annotations in the absence of independent sequence, catalytic, or structural evidence. For P1, representative predicted structures were compared with experimentally determined nucleoprotein structures from negative-sense RNA viruses. Structural superposition was performed using standard structural-alignment procedures, and conserved residues within structurally corresponding RNA-interacting regions were identified from the multiple sequence alignment and structural models. For P4, a representative AlphaFold2 model was compared with the vesicular stomatitis virus L protein structure PDB 5A22) [39], using structural superposition and Foldseek. The organization of the fingers, palm, and thumb subdomains of the RdRp core was assessed, and the positions of the canonical polymerase motifs were examined relative to the experimentally determined Mononegavirales polymerase architecture.

### 2.9. Functional Assessment of P2

Because conventional sequence searches failed to provide reliable functional annotation for P2, all 87 P2 structures were subjected to structure-based searches against the Protein Data Bank and AlphaFold Protein Structure Database using Foldseek. Putative functional assignments were classified according to the structural matches returned by Foldseek. Particular attention was given to structures related to the inosine triphosphate pyrophosphatase (ITPase)/Maf/HAM1 superfamily. The ITPase-like interpretation was independently evaluated using profile hidden Markov models. P2 sequences were searched against the Pfam Ham1 (PF01725) and Maf (PF02545) profiles using standard HMM-based search parameters. Human ITPA, Escherichia coli RdgB, and Saccharomyces cerevisiae Ham1 proteins were included as positive controls for the corresponding nucleotide-pool sanitization proteins. The presence or absence of significant profile matches was compared between the control proteins and the lispi-like P2 sequences. Sequence conservation within the P2 family was assessed from a multiple sequence alignment generated using MAFFT with standard parameters. Conserved residues were identified from the resulting alignment. For ITPase-like structures, residues corresponding to conserved catalytic or substrate-binding positions in characterized ITPase/HAM1 proteins were mapped onto the predicted structures. In particular, the structural equivalents of the conserved catalytic lysine were examined. Potential substrate-binding pockets in ITPase-like P2 models were inspected qualitatively by comparison with characterized ITPase/HAM1 structures. No molecular docking or quantitative ligand-binding calculation was performed. Structural observations were therefore interpreted as hypotheses concerning possible substrate recognition rather than evidence of enzymatic activity.

### 2.10. Analysis of P3 Topology and Structural Features

P3 proteins were examined as the third ORF in the conserved four-cistron genome organization. Predicted P3 structures were analyzed both as monomers and as homotrimers using AlphaFold2 and AlphaFold-Multimer, respectively. The predicted trimeric architecture was assessed for the presence of conserved helical regions, coiled-coil organization, and overall similarity to known viral fusion-protein architectures. Structural comparisons were performed using standard structural-alignment procedures. Potential membrane-associated features were assessed using Phobius, TMHMM, and SignalP 6.0. N-terminal signal peptides and transmembrane helices were recorded for each sequence. Potential N-glycosylation sites were identified from canonical N-X-S/T sequons, where X is any amino acid other than proline. Potential proteolytic cleavage motifs were assessed by searching the aligned sequences for conserved sequence patterns associated with known plant secretory-pathway proteases, using standard motif-based searches. The distribution of these features was evaluated across the complete 87-sequence dataset and within individual phylogenetic/genus-level groups.

### 2.11. Selection and Sequence-Conservation Analyses

Selection analyses of P3 were performed using codon-based alignments generated from the corresponding nucleotide and amino acid sequences. Protein alignments were used to guide the corresponding nucleotide alignment, and poorly aligned or ambiguous regions were excluded from downstream calculations. Synonymous and nonsynonymous substitutions were estimated using the Nei–Gojobori method [40]. Because synonymous substitutions were saturated across the full P3 dataset, dN/dS estimates were restricted to low-divergence sequence pairs for which synonymous divergence remained interpretable. Mean dN/dS values were calculated for the complete protein and for structurally defined regions, including the conserved coiled-coil and the more variable head/loop regions. Site-wise selection was assessed using within-cluster comparisons to reduce the effects of extreme sequence divergence and synonymous-site saturation. Codons were classified as showing evidence of purifying selection when the estimated synonymous/nonsynonymous substitution pattern met the statistical significance criterion used in the analysis; diversifying sites were identified using the corresponding criterion. Multiple-testing correction was applied where appropriate.

For P4, sequence conservation was quantified from the multiple sequence alignment using per-column amino acid entropy. Shannon entropy was calculated for each alignment position, with lower values indicating greater conservation. Conservation profiles were compared between the structurally resolved RdRp core and the more divergent C-terminal region using a one-sided Mann–Whitney U test.

### 2.12. Analysis of P4 Polymerase Motifs

The predicted P4/L proteins were examined for the canonical catalytic motifs of Mononegavirales RNA-dependent RNA polymerases. Conserved sequence blocks corresponding to palm motifs A, B, and C were identified from the multiple sequence alignment and compared with experimentally characterized Mononegavirales polymerases. Particular attention was given to the catalytic residues of motif A and the motif-C GDN signature characteristic of non-segmented negative-sense RNA virus polymerases. The conservation of these residues and their structural positions within the predicted RdRp core were assessed across all 87 P4 sequences. The structural organization of the P4 protein was compared with the L protein structure of vesicular stomatitis virus (VSV, PDB 5A22), with particular attention to the fingers, palm, and thumb subdomains and the relative position of the catalytic motifs.

### 2.13. Host-Association and Genus-Level Analyses

Host information associated with the viral sequences was obtained from the corresponding SRA/TSA metadata and source datasets. Host plant names were standardized to current taxonomic nomenclature and assigned to their corresponding botanical families. Virus–host associations were summarized at the species and family levels for each proposed viral genus. Host specificity was quantified as the number of distinct host species associated with each virus. Viruses detected in a single host species were classified as single-host viruses, whereas viruses detected in multiple host species were classified according to their observed host range. The distribution of host associations among proposed viral genera and plant families was visualized using standard statistical and graphical procedures. Where host-range distributions were compared between monocot-associated and dicot-associated groups, differences were evaluated using the Wilcoxon rank-sum test. Because transcriptomic datasets are unevenly sampled across host taxa, raw detection counts were interpreted together with the number of unique hosts, BioProjects, and other available metadata to distinguish repeated sampling from independent host associations.

### 2.14. Large-Scale Retrospective Search Using Logan

To assess the broader diversity and distribution of lispi-like viruses beyond the newly assembled sequences, publicly available RNA-sequencing libraries were screened using the Logan search platform [41]. The 87 newly identified lispi-like virus sequences, together with MSuV and the previously reported Disporopsis pernyi-associated lispi-like virus, were used as query sequences. Searches were performed against the publicly indexed RNA-seq library collection using the standard Logan search procedure and parameters. Positive libraries were retained when they contained significant sequence matches to one or more lispi-like virus queries according to the platform’s default detection criteria. For each positive library, associated metadata were retrieved where available, including host species, botanical family, geographic location, sequencing project, and library identifier. Duplicate library records were retained as individual sequencing observations but were distinguished from independent BioProjects when assessing sampling redundancy. The final dataset was used to summarize the number of positive RNA-seq libraries, unique host species, host families, and geographic locations. Detection frequencies were interpreted as observations of viral sequence presence in public sequencing libraries and were not treated as direct estimates of biological prevalence in natural populations.

### 2.15. Virus Prevalence and Host-Range Metrics

To account for differences in transcriptomic sampling intensity among host systems, a normalized Virus Prevalence Score (VPS) was calculated for viruses detected through the Logan search as VPS = (number of unique host species/√(number of positive RNA-seq libraries)) × 100. The square-root normalisation was used so that the index discounts, but does not fully cancel, differences in sequencing effort: dividing by the library count itself over-corrects and collapses heavily sampled viruses towards zero, whereas dividing by its square root retains a monotonic but damped dependence on sampling depth. Because the denominator is the square root of the library count rather than the library count, VPS is not a proportion and is not bounded at 100; values above 100 simply indicate viruses whose host diversity is high relative to the square root of their sampling effort. VPS is therefore a descriptive ranking index whose absolute magnitude has no interpretation independent of the dataset in which it is computed, and it is reported here only alongside the raw library counts and unique-host counts from which it is derived. The VPS was used as a descriptive normalization of the relationship between host diversity and the number of positive libraries. Raw library counts, unique host counts, and VPS values were considered jointly because each metric captures a different aspect of the available transcriptomic evidence. The number of independent BioProjects associated with each virus was additionally recorded to evaluate library redundancy and the extent to which repeated sequencing of the same biological system could inflate apparent detection frequency. Tissue-associated detections were classified according to the tissue metadata provided with the corresponding sequencing libraries. Tissue categories were standardized where possible, and the relative contribution of leaves, flowers, roots, stems, seeds, and whole-plant samples was summarized descriptively.

### 2.16. Assessment of Insect-Derived and Cross-Library Contamination

Because virus sequences recovered from plant transcriptomes could in principle derive from arthropods present on or within the sampled tissue, or from index hopping and cross-contamination between multiplexed libraries, the taxonomic composition of the source libraries was assessed independently of the viral assemblies. For each of the 67 independent BioProjects contributing the 87 genome sequences reported in Table 1, a representative sequencing run was retrieved and its precomputed SRA Taxonomy Analysis Tool (STAT) k-mer taxonomic profile was obtained through the NCBI Trace interface (https://trace.ncbi.nlm.nih.gov/Traces/sra-db-be/run_taxonomy, accessed on 30 July 2026). For each run, the numbers of reads assigned to Viridiplantae (NCBI taxid 33090), Arthropoda (taxid 6656), Insecta (taxid 50557) and Metazoa (taxid 33208) were expressed as percentages of all taxonomically identified reads in that run, and the ratio of plant-assigned to arthropod-assigned reads was calculated, using a pseudocount of one in each term to accommodate runs with no arthropod-assigned reads. STAT profiles were obtained for representative runs of 66 of the 67 BioProjects; the remaining project (PRJNA763316) has no precomputed STAT profile for any of its runs. In addition, the number of independent BioProjects, the number of distinct submitting groups and the range of deposition years underlying each virus were recorded, and the concordance between the L-protein phylogeny and host botanical family was evaluated as an internal test of biological, as opposed to technical, origin (Section 3.16).

## 3. Results and Discussion

### 3.1. Unveiling Plant-Associated Lispi-like Viruses

Recent findings on the only two plant-associated lispi-like viruses identified thus far indicate that these viruses do not induce discernible symptoms in their natural hosts [28,29]. This phenomenon has also been reported for other negative-sense plant viruses, such as varicosaviruses [42], cytorhabdoviruses [11], and nucleorhabdoviruses [43], suggesting that additional plant-associated lispi-like viruses might remain to be discovered in datasets derived from asymptomatic plants, where viral presence is not typically anticipated.

The advent of the powerful Serratus platform [8] has revolutionized the mining of this resource, enabling rapid and large-scale screening that would otherwise be prohibitively labor-intensive and time-consuming [11,12]. Leveraging this tool, we conducted the most extensive search to date on plant-associated lispi-like virus RdRp sequences. This effort led to the identification and assembly of full coding regions of 87 novel lispi-like viruses, including several variants associated with different plant hosts (Supplementary Table S2), increasing the number of known viruses in this group by 40×. Moreover, our findings significantly clarify their taxonomic position among the order *Mononegavirales*. These 87 viruses were associated with 75 plant hosts, where most of the apparent host plants are herbaceous dicots (57/75), while 18 hosts are monocots (Supplementary Table S2). Thus, these viruses likely have a host adaptation trajectory leading to preferentially infecting herbaceous dicots during their evolution.

### 3.2. Genomic Properties of Lispi-like Viruses

**Figure 1:**
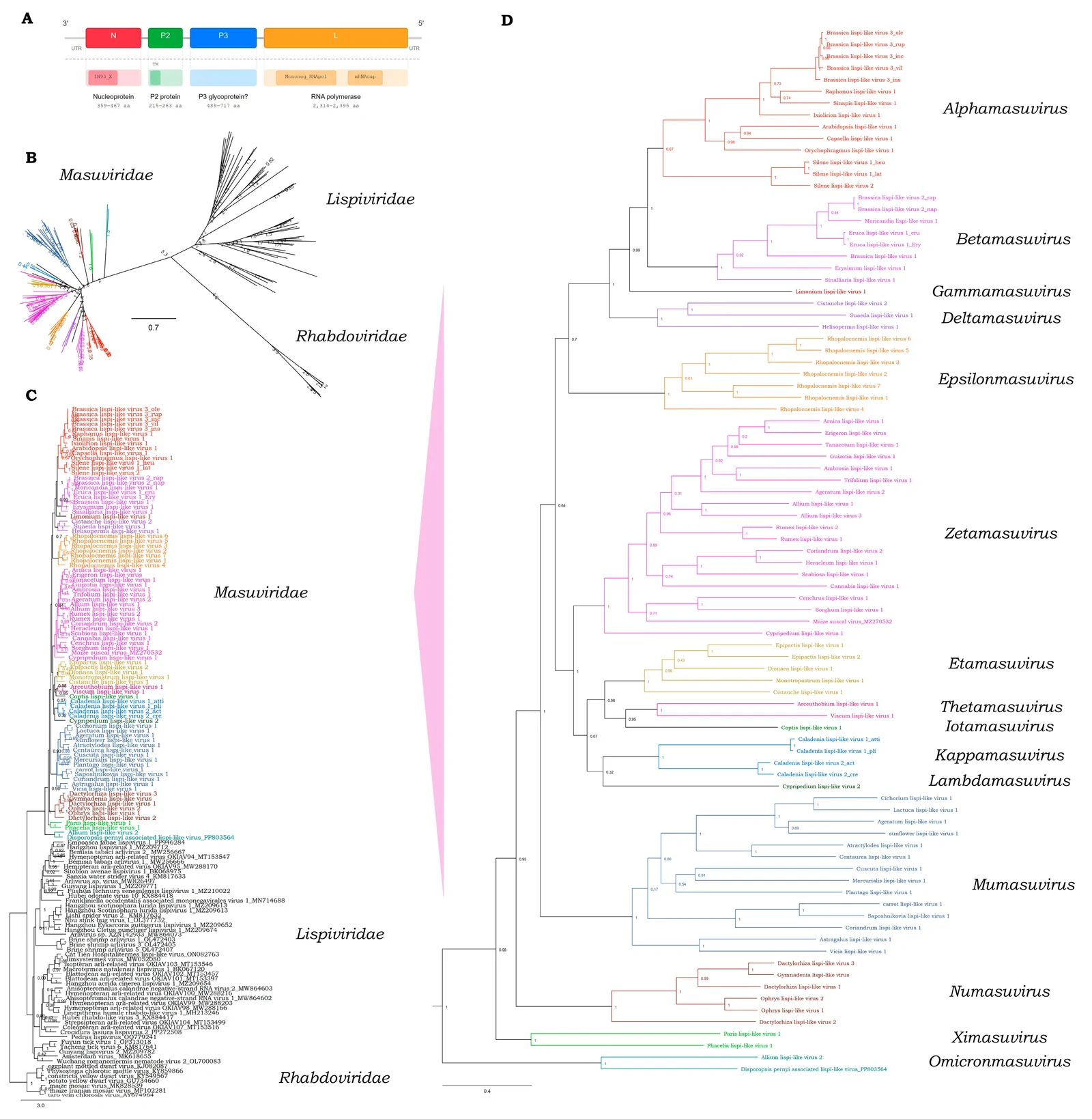
(**A**) Genomic organization of the viruses identified in this study. (**B**) Maximum-likelihood phylogenetic tree based on amino acid sequence alignments of the complete L gene of all reported so far and in this study, constructed with the WAG + G + F model (**C**,**D**). The scale bar indicates the number of substitutions per site. Bootstrap values following 1000 replicates are given at the nodes, but only the values above 50% are shown. Rhabdoviruses were used as the outgroup.

In contrast to other plant-associated mononegaviruses, such as the cytorhabdoviruses [11], among others, which display diverse genome organizations with many members having unique genome architectures [11,44], all novel lispi-like viruses display four genes in a single genomic organization 3′-N(P1)-P2-P3-L(P4)-5′ (Supplementary Table S2; Figure 1A), which was first reported for MSuV [28]. For clarity, and following the nomenclature used throughout this work, the first cistron is designated N (also referred to as P1) and the fourth is designated L (also referred to as P4); the two intervening cistrons, for which no functional assignment is implied by their names, are designated P2 and P3. This genomic conservatism suggests a homogeneous evolutionary trajectory, and its link to the family *Lispiviridae* tempts us to speculate that this novel group of viruses evolved from arthropod-infecting viruses and lost the G gene during its plant adaptation. This hypothesis is supported by the seed-transmission nature of MSuV [28], suggesting that these viruses are transmitted vertically. Further studies should focus on their potential vector, if any, and its mode of transmission.

The first ORF encodes the putative nucleocapsid, which is the protein commonly encoded by the first ORF in mononegaviruses [45]. The size of N proteins throughout the novel lispiviruses ranged from 359 to 467 aa (Supplementary Table S2), which is similar to the size range reported for counterparts from plant-associated mononegaviruses [26]. Moreover, BLASTP searches showed MSuV N protein as the best hit for each encoded N protein, but with a low identity value (Supplementary Table S2). The second ORF encodes a protein, named protein 2 (P2), and its size ranged from 215 to 263 aa. No similarity hits were found for this protein, even with relaxed parameters. Moreover, no conserved functional domains were identified in this protein, and no distant hits were found using HHblits or FoldSeek. Thus, further studies should be focused on the functional characterization of this protein to gain fundamental insights about its putative function. The third ORF encodes a protein, named protein 3 (P3), and its size ranged from 489 to 717 aa. As with P2, no similarity hits or conserved functional domains were found for this protein. The fourth ORF encodes the putative polymerase protein (L), and its size ranged from 2314 to 2395 aa, which is within the size range reported for L proteins from plant-associated mononegaviruses [26]. Moreover, BLASTP searches showed MSuV and Disporopsis pernyi associated lispi-like virus L proteins as the best hit for each encoded L protein, but sharing a low identity value (Supplementary Table S2); and lispivirus L proteins were the following best hits with identities below 30%. The Mononeg_RNA_pol, Mononeg_mRNAcap, and paramyx_RNAcap conserved domains were identified in each encoded L protein. These domains are conserved among the L proteins encoded by every mononegavirus [46].

The gene junctions of MSuV and of all newly identified viruses share a conserved, *Mononegavirales*-type architecture (Table 2). At the 3′ end of every gene, the mRNA-sense sequence terminates in a uridine-rich tract of five to seven residues preceded by an AU-rich segment (most commonly 3′-AU[C/A/U]UUUUUU), the expected signal for polymerase stuttering and mRNA polyadenylation in non-segmented negative-strand RNA viruses [46]. This termination signal is separated from the start of the downstream gene by an invariant trinucleotide intergenic spacer, most frequently CAC and less often CGC, and the downstream mRNA begins with a conserved trinucleotide, predominantly UCU or UCC. The three elements, terminator, spacer, and initiator, are conserved both among the masuviruses themselves and relative to MSuV, and their organisation parallels the compact, highly conserved gene junctions reported for other plant-associated mononegaviruses [11,42]. Moreover, the MSuV genome has complementary ends, which is a common feature of mononegaviruses [46]. Hence, the presence of a canonical U-rich termination/polyadenylation signal, a short conserved intergenic spacer and a conserved mRNA-start motif across the family provides independent, transcription-level support for the placement of this group within the order *Mononegavirales*, complementing the conserved genome organisation (Figure 1A) and the L-protein phylogeny (Figure 1B–D).

The inclusion of plant lispi-like viruses within the *Mononegavirales* is further supported by morphological properties observed for MSuV, which possesses rod-shaped virions [28], similar to those found for other plant-associated mononegaviruses [26].

### 3.3. Evolutionary Insights on Masuviruses

Pairwise aa sequence identities between L proteins belonging to the same species ranged from 88.08% to 99.45%. On the other hand, the pairwise aa sequence identities of L proteins across distinct species showed a great variation ranging from 29.92% to 79.98%, suggesting that there may still be an unknown amount of “virus dark matter” within plant-associated lispi-like viruses.

The phylogenetic analysis based on the L protein aa sequence showed that the 87 novel viruses, along with MSuV and Disporopsis pernyi-associated lispi-like virus, were grouped into a distinctive major group where four major clades could be distinguished (Figure 1B), and this group of viruses was linked to those members belonging to the *Lispiviridae* family (Figure 1B). This phylogenetic relationship indicates a common evolutionary history for this newly described group of viruses, where four clades of viruses showed distinct evolutionary trajectories.

Based on the phylogenetic insights and the observed genetic distance of the newly identified viruses, we tentatively propose an 80% aa sequence identity of the L protein as the threshold for species demarcation in each one on newly proposed genera, the 78 members for which the complete coding-sequence is available.

The phylogenetic analysis reveals that these plant-associated lispi-like viruses form a distinct monophyletic clade that diverges from the established family *Lispiviridae*, which comprises exclusively invertebrate-infecting viruses. This fundamental ecological transition between invertebrate and plant hosts, coupled with the substantial phylogenetic distance observed in RdRp-based trees, supports the establishment of a new viral family. The evolutionary trajectory evident in our analyses suggests an ancient host-switching event probably from arthropods to plants, followed by extensive diversification within plant hosts. This pattern of host realm transition accompanied by phylogenetic divergence parallels established criteria for family-level taxonomic distinctions in RNA virus classification. We therefore propose the family name *Masuviridae* (derived from Maize suscal virus, the first reported member of this lineage) to accommodate these plant-infecting viruses. The designation reflects both the historical precedence of maize suscal virus as the founding member and follows established viral nomenclature conventions. This taxonomic framework recognizes the fundamental biological differences between the invertebrate-associated *Lispiviridae* and their plant-associated sister group, while acknowledging their shared evolutionary origin within the broader lispi-like virus supergroup.

### 3.4. Structural Analysis of P1 Nucleocapsid Protein

**Figure 2:**
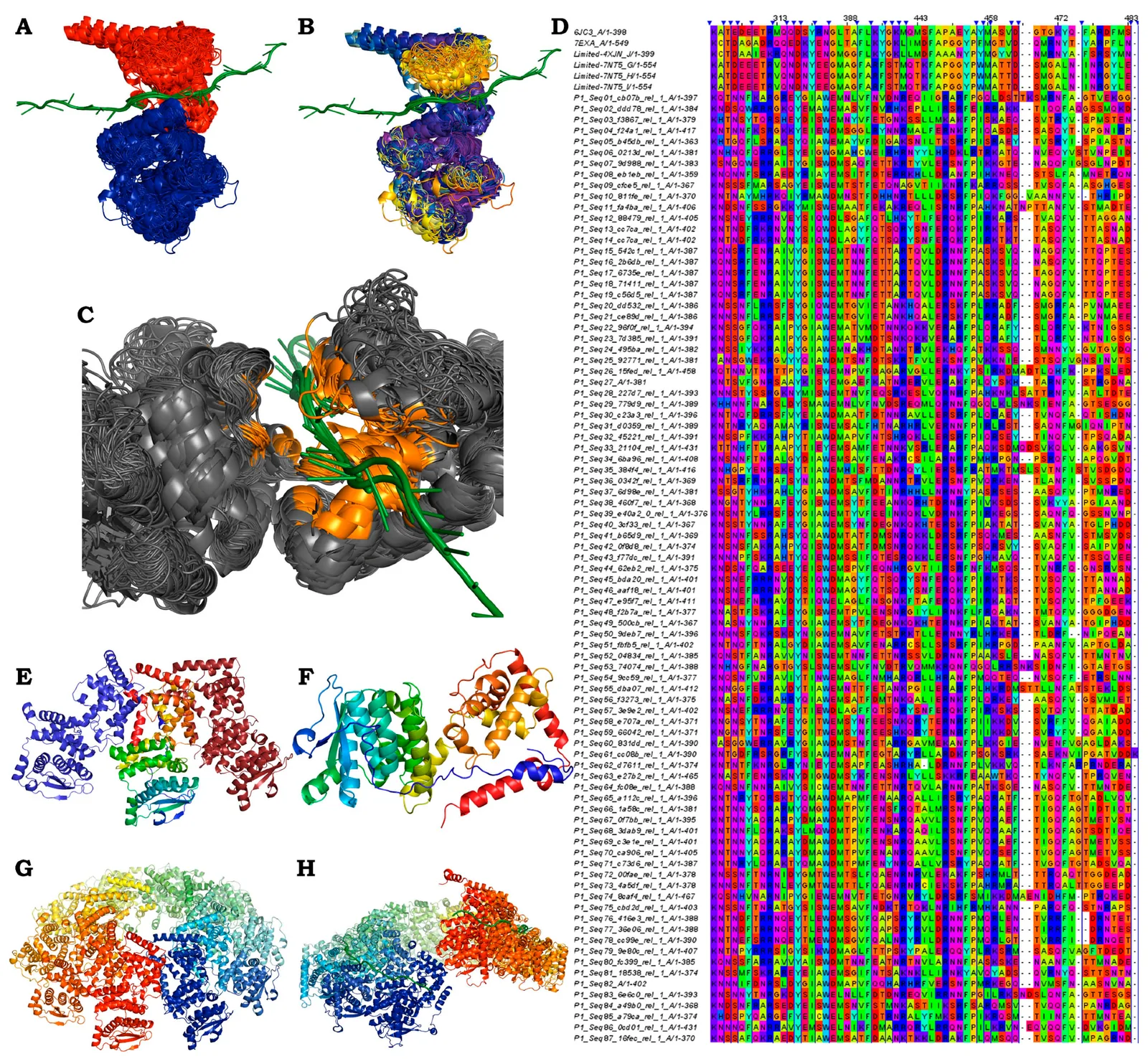
Structure of the P1 Nucleocapsid across Masuviridae shows consistent 5H + 3H structure containing groove with 3 up and 3 down nucleotides protected by a lid. (**A**–**C**) Structures of P1 superimposed by (**A**) domain [blue Nterm, red Cterm], (**B**) pLDDT from purple [100%] to red [0%], and (**C**) RNA-binding residues in orange, and shown as an (**D**) MSA. (**E**) Structural trimer of P1 shown with monomers on the sides in blue and red, while the central P1 is shown in spectrum from Nterm [blue] to Cterm [red], which is also shown in (**F**) profile. (**G**) 10 mer of P1 forming a circle and (**H**) 13 mer CryoEM structure of 7NT5 [7] from Nipah virus.

Computational structural modeling of P1 nucleocapsid proteins from 87 lispi-like virus sequences revealed a canonical two-domain architecture characteristic of negative-strand RNA viruses [47], comprising an N-terminal domain (NTD), flexible interdomain linker, and C-terminal domain (CTD) (Figure 2). This bipartite organization mirrors the conserved “5H + 3H” structural motif observed across the Negarnaviricota phylum [48,49], where nucleocapsid protein subunits are parallelly aligned along the RNA genome that is sandwiched between two domains composed of conserved helix motifs. AlphaFold2-based predictions showed high confidence for core functional domains (pLDDT > 90) with excellent structural superposition against six experimentally determined nucleoprotein structures (PDB IDs: 6JC3, 4XJN, 1N93, 5WKN, 7EXA, 7NT5), while lower confidence in terminal regions (pLDDT 50-70) reflected expected intrinsic disorder in regulatory domains.

The NTD exhibits a complex α/β architecture (β1-L1-α1-L2-α2-L3-α3-L4-β2-L5-α4-L6-α5-L7-α6-L8-α7-L9-α8-L10-α9-L11-α10) with critical RNA-binding residues showing exceptional conservation: K164 in loop L7 (100% conservation), R178 in helix α6 (99% as Arg/Lys), and Y242 in helix α10 (94% conservation). The interdomain linker contains highly conserved residues essential for RNA binding, including W249 (89% conservation) for aromatic stacking interactions and E251 (58% Glu, 34% Asp) for electrostatic RNA backbone contacts. The all-α-helical CTD (α1-L1-α2-L2-α3-L3-α4-L4-α5-L5-α6-L6-α7-L7-α8-L8-α9) features universally positively charged residues R299 (67% Arg, 26% Lys) and R312 (82% Arg, 12% Lys) for RNA phosphate interactions, alongside aromatic residues F257 (84%) and F315 (84% Phe, 15% Tyr) for nucleobase recognition.

This conservation pattern parallels observations in related virus families where no consensus interactions with the bases of the encapsidated RNA were identified among all reported nucleocapsid-like particle structures [47,48], suggesting that nucleocapsid proteins must accommodate all possible RNA sequences. The amino acid substitution patterns follow chemically conservative changes (Phe ↔ Tyr, Arg ↔ Lys), indicating that functional constraints, rather than phylogenetic relationships, drive sequence evolution at RNA-binding sites. Notably, regions α8, L7, and L8 in the CTD show high structural variability, suggesting adaptive mechanisms for accommodating diverse RNA sequences across different lispi-like viruses, similar to the protein plasticity enabling non-sequence-specific interactions observed in Nipah virus nucleocapsid assembly [50] (Figure 2).

Structural analysis reveals that N protomers encapsidate RNA following the “3-bases-in, 3-bases-out” geometric arrangement with six nucleotides per protein monomer, consistent with paramyxovirus nucleocapsid assembly [50]. This packaging model predicts 1500–2000 N protomers are required for complete genome encapsidation (9000–12,000 nucleotides), representing highly efficient packaging that maximizes RNA protection while minimizing protein investment. The proposed helical ribonucleoprotein complex forms through inter-protomer contacts, creating continuous RNA-binding channels, enabling accommodation of variable genome lengths while maintaining consistent structural interactions (Figure 2).

The universal conservation of critical residues (K164, R178, W249, R312) demonstrates strong selective pressure to maintain specific RNA-protein contacts essential for genome packaging, aligning with the hallmark characteristic of negative-strand RNA viruses where genomes never exist as free RNA but are always assembled with nucleoproteins to form highly stable nucleocapsids. The near-universal conservation of W249 for RNA stacking and the conserved positive charge distribution (R178, R299, R312) present attractive targets for broad-spectrum antiviral development, as nucleocapsid-disrupting agents targeting these interactions could demonstrate pan-lispi activity.

While computational predictions show excellent agreement with experimental structures for core domains, experimental validation is still required to confirm those predictions. The low-confidence terminal regions likely reflect genuine intrinsic disorder consistent with regulatory functions, and static models cannot capture dynamic conformational changes during RNA binding and assembly. The proposed “3-bases-in, 3-bases-out” packaging model, while consistent with related nucleoproteins, represents one interpretation requiring validation through cryo-electron microscopy of complete nucleocapsid particles. Alternative packaging arrangements, including asymmetric RNA binding or variable stoichiometry models, should be considered alongside experimental mutagenesis targeting highly conserved residues to definitively establish structure–function relationships and validate the helical assembly mechanism proposed from our structural analysis.

### 3.5. Structural Analysis of P2

#### 3.5.1. Structure-Based Functional Annotation

To probe the function of the P2 protein, whose primary sequence is too divergent for reliable annotation by conventional sequence-homology search, we generated AlphaFold2 models for all 87 lispi-like virus P2 sequences and queried each predicted structure against the Protein Data Bank and the AlphaFold Database using Foldseek. This structure-based strategy exploits the fact that protein folding is considerably more conserved than the aa sequence [51], allowing remote homologies to be detected where the sequence signal has decayed [33].

**Figure 3:**
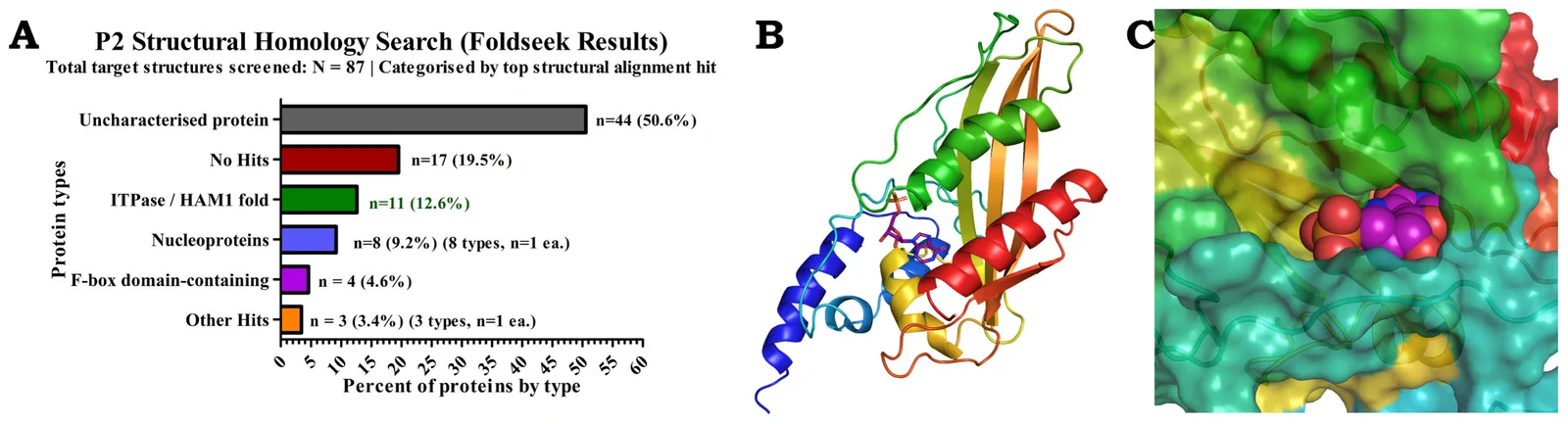
P2’s diversity (13.9% mean pairwise amino-acid identity; median 12.0%) leads to only 11 structures labelled as ITPases. (**A**) P2 Foldseek results across the 87 masuviruses. Structure of P2 in ITPase protein structure shown with IMP [purple] in the catalytic pocket (**B**) as a cartoon and (**C**) with surface structure coloured in rainbow spectrum Nterm [red] to Cterm [blue].

The dominant outcome was an absence of interpretable annotation. Sixty-one of the 87 models (70%) returned no usable functional assignment: 30 matched only proteins that are themselves annotated as “uncharacterized”, 17 produced no significant structural hit, and 14 yielded matches too inconsistent or too low in confidence to interpret (Figure 3). P2 therefore belongs largely to the poorly characterized “dark” fraction of the viral proteome, in sharp contrast to the strong, internally consistent annotation obtained for the putative nucleocapsid from the same isolates.

Of the 26 models (30%) that did return a putative assignment, the single most frequent was inosine triphosphate pyrophosphatase (ITPase; Maf/HAM1 family), recovered for 11 sequences, roughly 42% of all annotated models and 13% of the full set. The remaining assignments were largely singletons spanning nucleotide- or nucleic-acid-associated activities (an ATP/GTP-binding protein, a cytosine/adenosine deaminase, GTPases, a valyl-tRNA ligase, a ribonuclease, a putative RNA-binding protein, and a zinc-finger), together with an F-box-related group and a small number of non-nucleotide annotations. In all, approximately three-quarters of the interpretable hits (19 of 26) corresponded to functions that act on nucleotides or nucleic acids; the dominant and most coherent signal is nucleotide metabolism, specifically purine-pool sanitization, rather than direct nucleic-acid binding.

The predicted P2 architectures were themselves structurally heterogeneous. Most models adopted a mixed α/β fold organized around a small central β-sheet (commonly two to five strands) flanked by α-helices; a substantial minority were entirely α-helical; a smaller subset displayed two distinct β-sheets; and four models (sequences 22, 33, 35 and 66) showed no discernible secondary-structure organization. The recurrent mixed α/β topology corresponds to the canonical architecture of the ITPase/HAM1/Maf superfamily, whereas the all-α subset is reminiscent of the distinct all-α (MazG-type) NTP-pyrophosphatase superfamily; both are among the four recognized structural classes of nucleotide “house-cleaning” enzymes [52]. For the ITPase-annotated models, qualitative inspection of cavity geometry, not automated docking, indicated a pocket dimensionally complementary to inosine that would sterically exclude the exocyclic N6 amino group of adenosine while leaving room for xanthine, a pattern mechanistically coherent with ITPase substrate selectivity (Figure 3). We stress that this is a static, qualitative observation presented as a structural prediction rather than evidence of catalytic activity.

#### 3.5.2. Sequence-Level Properties and Independent Tests of the ITPase Signal

The 87 P2 proteins form a single, full-length protein family: all begin with methionine, and their lengths occupy a narrow window (211–273 residues; median 231). Despite this coherence, the family is extraordinarily divergent, with a mean pairwise amino-acid identity of 13.9% (median 12.0%; range 3.1–99.6%), a value below the twilight zone of reliable sequence-based homology inference [53]. A multiple-sequence alignment recovered only a vestigial conserved core: of 217 well-populated columns, just 17 are conserved in at least half of the sequences and only four exceed 70% conservation, comprising fold-scaffold residues including a near-invariant tryptophan and lysine and a few buried hydrophobic and Gly/Pro positions. The heterogeneous structural annotations therefore reflect divergence and prediction noise within one protein family rather than a mixture of unrelated proteins.

Against this backdrop, we tested the ITPase assignment with the most sensitive sequence-level methods available. Profile hidden Markov models for the Pfam Ham1 (PF01725) and Maf (PF02545) families [54,55] detected the three reference ITPases used as positive controls, human ITPA, *Escherichia coli* RdgB, and *Saccharomyces cerevisiae* Ham1, at high significance (176–223 bits; E ≤ 1 × 10^−54^), yet returned no hit (E < 10) for any of the 87 P2 proteins, including all 11 that Foldseek had flagged as ITPase. Consistent with this, the flagged proteins shared only 4–10% identity with the reference ITPases in direct alignment, and the catalytic lysine that contacts the substrate triphosphate (human ITPA K19, equivalent to UCBSV Ham1 K38; 56) was conserved in just 2 of the 11. The ITPase signal is thus confined to the structural level and is not corroborated by sequence or profile homology.

The flagged proteins also do not constitute a coherent sequence lineage: their mean within-group identity (18.6%) is only marginally above their identity to the rest of the family (14.1%), and they disperse across a sequence-similarity clustering rather than forming a clade. They are, however, structurally self-consistent, with 10 of the 11 falling in the mixed α/β class. Physicochemically, the family is near-neutral (mean isoelectric point ≈ 7) and slightly hydrophobic, and is not enriched in basic residues; the flagged subset is indistinguishable from the remainder on these measures. This composition is consistent with a globular enzyme and argues against a primary, basic-residue-driven nucleic-acid-binding role. Finally, the four models lacking discernible secondary structure are compositionally ordinary globular proteins by the charge–hydropathy criterion [56] rather than predicted to be intrinsically disordered, indicating that these cases most plausibly reflect low-confidence prediction rather than genuine disorder (Figure 3).

Mapping each isolate to its host yielded a broad plant range dominated by *Brassicaceae*, *Asteraceae*, *Orchidaceae* and *Apiaceae* and including several parasitic or mycoheterotrophic plants. Structural class showed no association with host family, and the 11 ITPase-flagged viruses spanned seven plant families with no representative of the *Euphorbiaceae*, the family of all four RNA viruses previously reported to encode a Maf/HAM1 domain [57]. The single *Euphorbiaceae*-infecting isolate in our set (*Mercurialis* lispi-like virus 1) returned no structural hit for P2. The host-linked pattern that characterizes the cassava system is therefore not reproduced here. We further note several tight within-species strain clusters (in the *Brassica*, *Caladenia*, *Eruca* and *Silene* virus groups); these introduce statistical non-independence and were treated as a caveat for any host-correlation or selection analysis.

The most prominent and internally consistent structural signal to emerge from this analysis is the recurrent assignment of a subset of P2 proteins to the ITPase (Maf/HAM1) family. ITPases are house-cleaning enzymes conserved across all domains of life that hydrolyze deaminated, mutagenic purine nucleotides, chiefly ITP, dITP, XTP and dXTP, to their monophosphates, preventing their misincorporation into RNA and DNA and thereby limiting mutation [58]. Their presence in RNA-virus genomes is rare: plant RNA viruses are under strong selection for compact genomes, and to date ITPase/Maf-HAM1 domains have been reported in only four RNA viruses, all infecting *Euphorbiaceae*, three potyvirids (cassava brown streak virus, Ugandan cassava brown streak virus, and euphorbia ringspot virus) and one secovirid, cassava torrado-like virus [56]. A genuine ITPase-like P2 in lispi-like viruses, if confirmed, would represent a phylogenetically independent and far broader occurrence of this unusual viral accessory function, which is precisely why the evidential basis for the assignment must be weighed carefully.

Experimental work on the cassava brown streak viruses provides the closest functional template. Their Ham1 proteins possess genuine in vitro ITPase activity with a preference for the non-canonical substrates dITP and XTP, act as determinants of necrotic symptoms [59], and are required for infectivity in cassava but dispensable in the model host *Nicotiana benthamiana*, a host-specific dependence attributed to the unusually high cytoplasmic concentration of non-canonical nucleotides in cassava, with the viral enzyme acting in partnership with the viral RNA-dependent RNA polymerase [56]. Under this model, a virus-encoded ITPase lowers the effective mutational load on the replicating genome, with the benefit, and therefore retention of the domain, contingent on the host’s nucleotide chemistry.

Our data, however, place clear limits on how far this template can be extended to P2. The structural evidence and the sequence evidence point in different directions. On the structural side, the ITPase assignment recurs across 11 models; those models reproduce the central-sheet-flanked-by-helices architecture of the ITPase/HAM1/Maf superfamily, and the predicted cavity is geometrically complementary to inosine, three observations that are mutually consistent. On the sequence side, the same proteins carry no detectable Ham1 or Maf profile signature, retain the catalytic lysine in only a minority of cases, do not form a coherent sequence group, and show no host association with *Euphorbiaceae*. The divergence of the family (mean identity ≈ 14%, below the twilight zone) means that the absence of a sequence signal is not by itself decisive, because fold is conserved more deeply than sequence [51]; a true but ancient homology could in principle have erased the profile signal. Even so, the negative result is informative, because the Ham1 profile readily recovers ITPases from bacteria, fungi and humans, and the failure to detect even a weak match across 87 proteins is a real constraint. The defensible conclusion is that nucleotide-pool sanitization remains the leading functional hypothesis for the ITPase-like subset, but that it now rests on structural evidence alone and should be presented as such.

Several alternatives merit explicit consideration. First, because Foldseek can return confident geometric matches between analogous but non-homologous folds, and because the flagged proteins are dispersed in sequence rather than forming a lineage, a fraction of the ITPase assignments may reflect structural convergence or model noise rather than homology. Second, the breadth of minor annotations and the structurally distinct all-α subset suggest that P2 may be a rapidly evolving, modular or multifunctional accessory locus rather than a single conserved activity. Third, even where an ITPase-like fold is genuine, catalytic competence is not guaranteed: the patchy conservation of the catalytic lysine is consistent with a structurally intact but catalytically degraded “pseudo-ITPase” in at least some isolates. These possibilities are not mutually exclusive, and distinguishing them is the central question for future work on P2.

These conclusions rest on computational structure prediction and on sequence and profile search, and several limitations bound their interpretation. AlphaFold2 confidence was not uniform across models, and the no-fold and highly variable cases likely include low-pLDDT artefacts that inflate the apparent structural diversity; per-residue confidence should be reported alongside each model, and the four “no-structure” cases in particular reinterpreted as low-confidence predictions rather than disordered proteins. The inosine-pocket assessment is static and qualitative. The decisive remaining tests are therefore structural and biochemical rather than sequence-based: reading the residue structurally equivalent to ITPA K19/Ham1 K38 directly from each predicted model; a profile–profile search (for example HHpred) as a final remote-homology check; and, definitively, an enzymatic assay of recombinant P2 against ITP and XTP versus canonical NTPs, ideally with a co-crystal or cryo-EM structure of an enzyme–substrate complex. A test of selection (dN/dS) on a codon alignment, and a reconciliation of the P2 phylogeny with the host and RNA-dependent RNA polymerase trees, would establish whether the ITPase-like variants are monophyletic and whether their retention tracks host taxonomy as in the cassava system; both require nucleotide and reference-tree data not analyzed here, and any such analysis should first collapse the within-species strain clusters identified above. Until then, the most defensible statement is that a structurally coherent subset of lispi-like virus P2 proteins resembles the ITPase/HAM1 family and presents an inosine-complementary cavity, making nucleotide-pool sanitization a plausible but unconfirmed function, while the majority of P2 sequences remain functionally uncharacterized and the family as a whole is too divergent for sequence-based annotation.

### 3.6. Structural Analysis of P3

**Figure 4:**
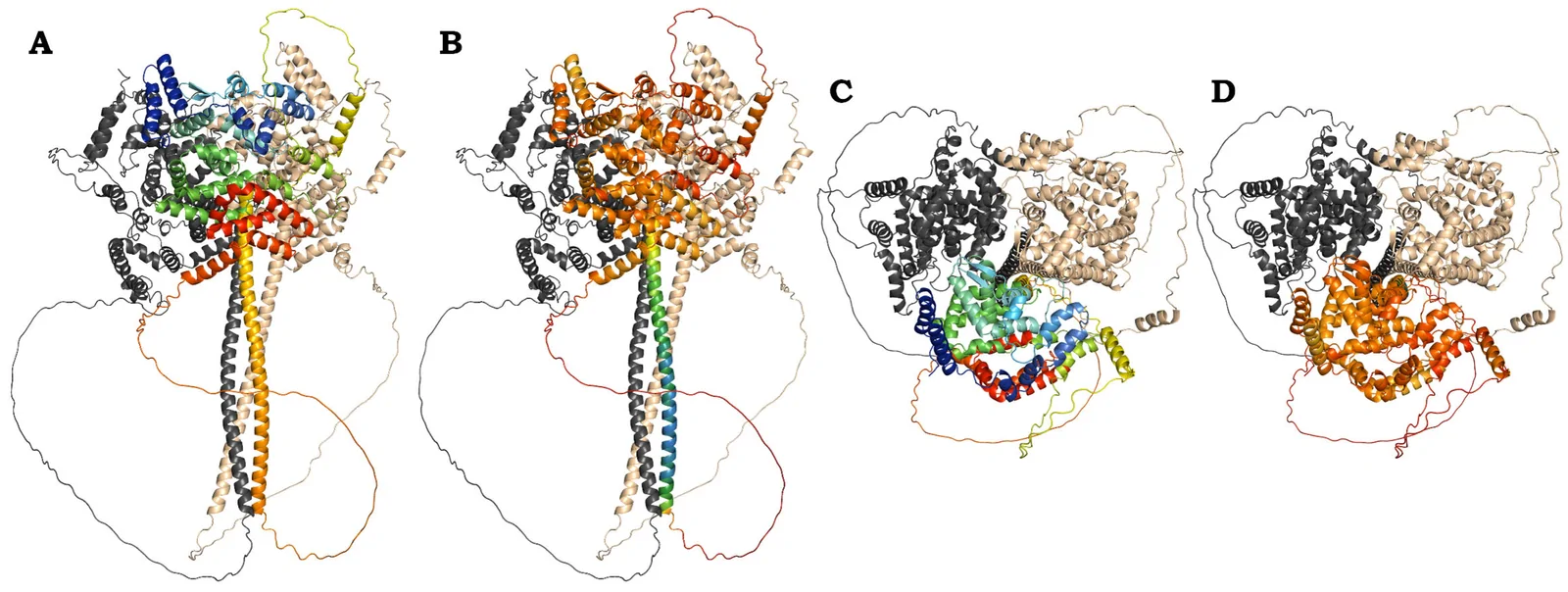
52/87 P3 proteins formed a glycoprotein with a confident coiled-coil but an uncertain head region resembling post-fusion proteins. (**A**) P3 trimer shown from (**A**,**B**) the side and (**C**,**D**) the top, with chain A of each denoting a rainbow spectrum of (**A**,**C**) Nterm [blue] to Cterm [red] or pLDDT confidence (**B**,**D**) from purple [100%] to red [0%].

The third major open reading frame (ORF3, encoding the protein termed P3) occupies the genomic position that, in the invertebrate-infecting *Lispiviridae*, encodes the viral glycoprotein, and a genome-wide alignment confirmed that all 87 viruses share this architecture. Consistent with extreme divergence, P3 returned no hits in BLASTP or PSI-BLAST searches, so it was characterised by structure prediction and by model-independent sequence analysis. AlphaFold2 modelling produced an essentially unstructured monomer, but assembly as a homotrimer yielded, for 54 of the 87 sequences, an elongated particle reminiscent of a class-I viral fusion protein in its post-fusion conformation, a globular head subtended by a long helical stalk, visually comparable to the post-fusion foamy virus envelope glycoprotein [60]. The confidence of these models was strongly non-uniform: the stalk helices modelled at moderate confidence (pLDDT ≈ 50–80), whereas the globular head fell below 40, that is, was effectively undetermined (Figure 4).

At the sequence level, the 87 P3 proteins form a single, full-length family, all begin with methionine, and lengths occupy a coherent window (489–717 residues, mean 624), roughly 300 residues shorter than the foamy virus glycoprotein precursor. The family is nonetheless hyper-divergent, with a mean pairwise amino-acid identity of 13.3%, below the twilight zone of reliable sequence-homology inference [53], which accounts for the failed similarity searches. Against this low background, one feature is conserved family-wide: every sequence carries a sustained heptad-repeat coiled-coil, and the coiled-coil register is positionally conserved across the alignment, with all 87 sequences in register at the most conserved column. This conserved coiled-coil is the element that forms the trimeric stalk in the models and is, by a wide margin, the most strongly constrained part of the protein (Figure 4).

The membrane-protein hallmarks expected of a surface glycoprotein are, however, absent at the family level. Combined signal-peptide and transmembrane-topology prediction (Phobius; 32) assigned a signal peptide to only 6 of 87 sequences, five of which constitute a single near-identical *Brassica* strain cluster, and a transmembrane segment to just one, so the large majority are predicted to be cytoplasmic, with neither an N-terminal secretion signal nor a C-terminal membrane anchor. The proteins are cysteine-poor (mean ≈ 5 residues, ≈ 0.7%), unlike the disulphide-stabilised ectodomains of characterised fusion glycoproteins, and N-glycosylation sequons occur at approximately the frequency expected by chance (≈3.7 per protein) with no sequon conserved across the family. A substantial fraction of each protein is predicted to be intrinsically disordered (mean ≈ 30% of residues, up to ≈68% in individual sequences), concentrated in the head and in long inter-domain loops, the same regions that modelled at low pLDDT.

Because P3 is encoded as a standalone cistron rather than within a polyprotein, any proteolytic maturation would have to be carried out in trans by host proteases. Scanning for plant-relevant cleavage motifs, the Site-1-protease/SKI-1 ortholog (SBT6.1/AtS1P), vacuolar legumains/VPEs, and, for completeness, furin-type sites (noting that plants lack the metazoan furin convertases), recovered no motif positionally conserved across the family, and no candidate located at the head–stalk junction ahead of a hydrophobic, fusion-peptide-like segment. When conservation was instead assessed within the 11 multi-member, host-coherent groups recoverable from this sparsely sampled, roughly 13–15-genus family (mean within-group identity 67%), cluster-level conserved sequons and S1P-type motifs did emerge, candidates that a family-wide scan necessarily discards. Their interpretation is bounded on two counts: the short S1P-type motif is frequently shared within a group by descent rather than by selection, and the relevant plant proteases, like N-glycosylation itself, act only on proteins that enter the secretory pathway, which here is restricted to the two signal-peptide-bearing lineages (*Brassica* and *Limonium*).

Selection analysis on a codon alignment confirmed that P3 is a genuine, functionally constrained gene. Family-wide, synonymous sites are saturated (proportion of synonymous differences ≈ 0.75), so global rate ratios are uninterpretable; estimated within low-divergence pairs, the protein is under clear purifying selection (dN/dS ≈ 0.10), and the constraint is spatially structured, the coiled-coil core being markedly more constrained than the head and loops (dN/dS ≈ 0.08 versus ≈0.24). Site-wise analysis using within-cluster comparisons classified 61% of testable codons as significantly purifying and none as diversifying. The variable head is therefore under relaxed purifying selection rather than positive selection, and shows no signature of the antigenic or receptor-driven diversification often seen in the receptor-binding heads of fusion glycoproteins.

Three lines of evidence place P3 as the plausible, if highly divergent, positional homolog of the *Lispiviridae* glycoprotein. It occupies the syntenic glycoprotein cistron across all 87 genomes; it assembles into an elongated homotrimer whose architecture resembles a post-fusion class-I fusion protein [60,61]; and its single conserved, family-wide structural element is a coiled-coil that forms the trimeric stalk, the heptad-repeat coiled-coil being the defining core of class-I fusion machinery [62]. The selection landscape reinforces this reading at the level of architecture, in that the coiled-coil core is the most strongly purifying-selected region while the head is variable, paralleling the conserved fusion core and variable head of characterised fusion proteins.

Against this, the features that define a *matured surface* glycoprotein are absent family-wide. Class-I fusion glycoproteins are synthesised with an N-terminal signal peptide, anchored by a C-terminal transmembrane domain, N-glycosylated on a disulphide-stabilised ectodomain, and proteolytically primed by a host protease [61,62]. P3, as predicted from sequence, has none of these as a conserved property: no signal peptide or transmembrane anchor in the large majority, few cysteines, chance-level glycosylation, and no family-wide cleavage site. Because the family is so divergent that synonymous sites are saturated and identity lies below the twilight zone, the absence of these signals cannot by itself exclude a fusion glycoprotein; divergence can erode or relocate signal peptides, anchors, and cleavage sites, but neither do the data positively support a canonical, membrane-displayed, glycosylated, primed glycoprotein. The defensible reading is that the conserved coiled-coil and the trimeric model are real and fusion-protein-like, whereas the maturation and membrane-targeting apparatus expected to accompany them is not detectable at the family level.

The strength of the structural impression also warrants caution about its origin. AlphaFold-Multimer readily generates plausible, symmetric, elongated coiled-coil assemblies, and a model combining a confident central coiled-coil with a low-confidence peripheral domain is a recognised failure mode rather than necessarily a biological structure [38]. Here the head is modelled below 40 pLDDT, effectively unmodelled, and three independent measures (pLDDT, predicted disorder, and relaxed selection) agree that this region is poorly determined. The post-fusion resemblance therefore rests on the conserved coiled-coil, the synteny, and the trimerisation, none of which establishes a fusion function on its own. Quantitative structural comparison, for example, Foldseek or TM-align against experimentally determined post-fusion fusion proteins [33], together with interface-confidence metrics (ipTM, predicted aligned error), would be required to test the resemblance properly.

The diversity of the family shapes how its functional features should be read. With members spanning on the order of 13–15 prospective genera, candidate functional sites are not expected to be conserved across all 87 sequences, and indeed glycosylation sequons and S1P-type cleavage motifs are conserved within genera even though none is conserved family-wide, a pattern that a whole-family conservation test would erroneously read as absence. Whether any of these represents genuine, used machinery is gated by subcellular localisation: only the two secretory lineages could be glycosylated or processed by secretory plant proteases, so genus-level processing, if it occurs, is likely lineage-specific rather than a family-wide property. Beyond a divergent fusion protein, the data remain compatible with alternative roles for a conserved trimeric coiled-coil protein at this genomic position, such as a structural or scaffolding function; and the absence of diversifying selection argues specifically against a classical antigenic receptor-binding head, though not against a structured head that simply was not modelled.

Several limitations bound these conclusions. The structural inferences rest on AlphaFold models whose interface confidence was not evaluated here and whose head is undetermined; the topology, glycosylation and cleavage predictions are sequence models that may miss non-canonical determinants in a divergent viral protein; selection was estimated by Nei–Gojobori counting [40] rather than by a maximum-likelihood site model, so the count of purifying sites is a lower bound and the absence of diversifying sites should be confirmed per genus with a likelihood method [63]; and cluster-level conservation partly reflects shared ancestry. Resolving the identity of P3 will require experiments, N-terminal sequencing or mass spectrometry of virion-associated P3 to detect maturation, glycosidase and membrane-fractionation assays to test glycosylation and membrane association, and ultimately a determined trimer structure. Until then, the data read most defensibly as a conserved, trimer-forming, coiled-coil protein occupying the glycoprotein cistron and under strong purifying selection on its core, carrying a structurally undetermined, disordered, lineage-variable head, and lacking the family-wide hallmarks of a matured surface glycoprotein.

### 3.7. Structural Analysis of P4

#### 3.7.1. The Largest Lispi-like Virus ORF Encodes an L-Protein-like RdRp

The fourth open reading frame encodes the large (L) protein, hereafter also P4, which is by a wide margin the largest cistron in the lispi-like virus genomes; its 87 translated products are strikingly uniform in length (2314–2402 aa; mean 2348 aa, s.d. 18 aa). This size and the collinear arrangement of conserved blocks are characteristic of the multidomain L protein of the *Mononegavirales*, which integrates the RNA-dependent RNA polymerase (RdRp), the polyribonucleotidyltransferase (PRNTase) capping activity, and a cap methyltransferase within a single polypeptide [39,46,64]. Because the RdRp is the single most conserved and most taxonomically diagnostic protein across RNA viruses, and the primary character used by the ICTV to delimit *Mononegavirales* families [25,65], we examined P4 both structurally and at the sequence level to test its identity and its catalytic competence.

#### 3.7.2. The Structural Model Recovers a Canonical RdRp Core but Not the Accessory Domains

**Figure 5:**
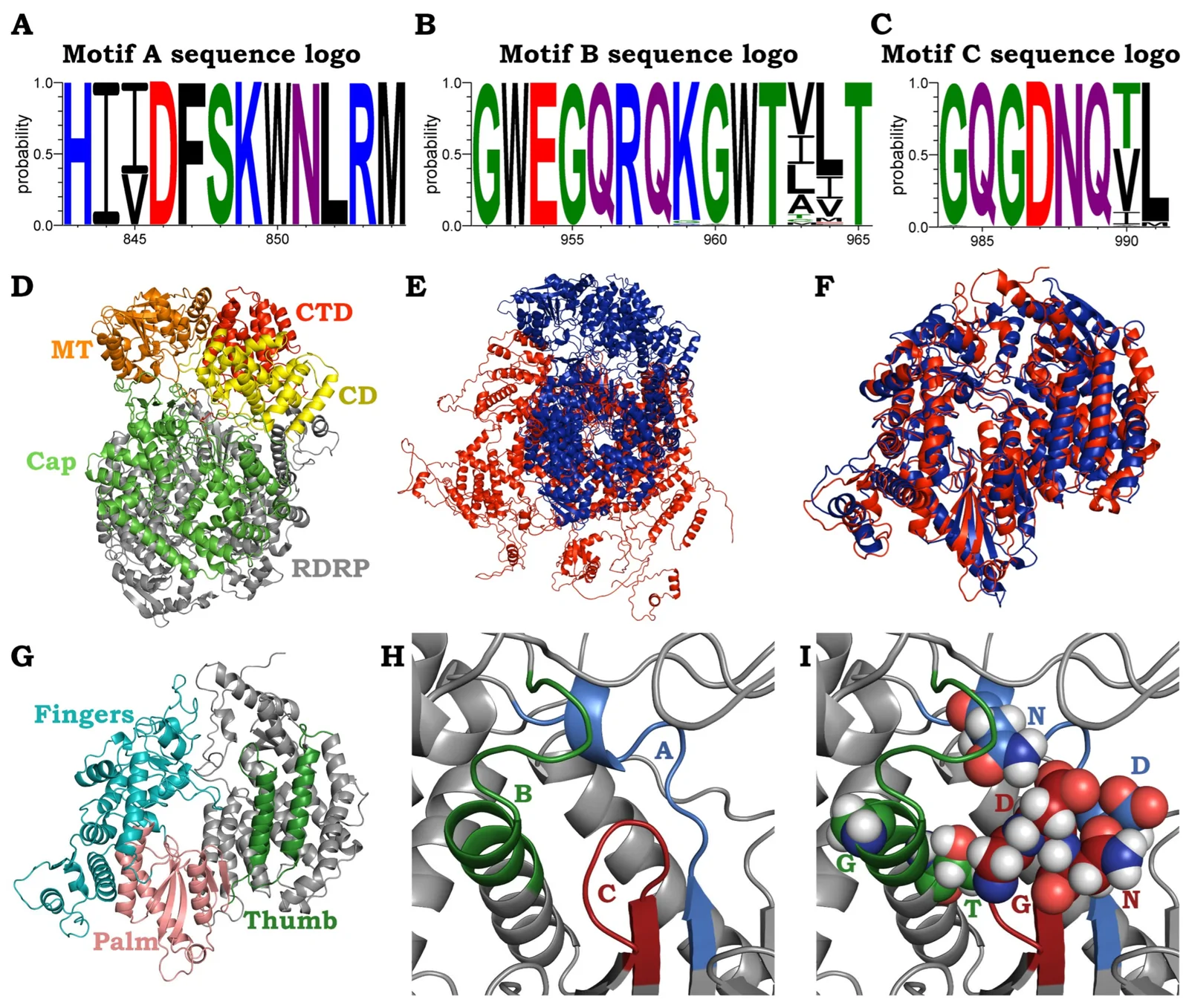
P4 of Masuviridae maintains core RDRP and catalytic palmprint, but surrounding domains were not resolved. (**A**–**C**) Sequence logos of the 3 core motifs of RDRP. (**D**) Structure of VSV RDRP with all domains from 5A22 [8]. (**E**,**F**) Structure of P4 and the core RDRP [red] compared to VSV [blue]. (**G**) RDRP of P4 shown highlighting the Thumb, Palm, and Fingers of the core structure. (**H**) Zoomed view of the Palmprint motifs of P4 RDRP with (**I**) key residues highlighted.

An AlphaFold2 model of a representative P4 [37] was compared with the cryo-EM structure of the vesicular stomatitis virus (VSV) L protein (PDB 5A22; 39), the prototypical *Mononegavirales* polymerase, using structural superposition and Foldseek [33] (Figure 5D–F). The comparison resolves cleanly into two regimes. The N-terminal RdRp module of P4 superimposes on the VSV RdRp core (Figure 5E,F, core in red) and reproduces the canonical right-hand polymerase architecture, with clearly delineated fingers, palm and thumb subdomains (Figure 5G). Within the palm, the three catalytic palmprint motifs A, B and C [64,66] are present and correctly positioned (Figure 5H), and the key metal-coordinating and template-contacting residues project into the active-site cleft in the expected geometry (Figure 5I). By contrast, the domains that flank the RdRp in a complete L protein, the PRNTase/capping domain, the connector domain, the methyltransferase and the C-terminal domain (Figure 5D), did not superimpose confidently onto their VSV counterparts and were not resolved as discrete, VSV-like folds in the model. The structural evidence therefore supports a bona fide RdRp core embedded in a large L-like protein whose accessory modules could not be confidently modelled by homology.

#### 3.7.3. P4 Is Markedly More Conserved than the Other Lispi-like Virus Proteins, and Its Divergence Is Structured by Host Family

Across the 87-sequence alignment, P4 shows a mean pairwise amino-acid identity of 39.9% (median 38.5%, range 29.0–99.5%; Supplementary Figure S1A). These pooled values are consistent with the partitioned identities reported in Section 3.3, where the same L-protein alignment yields 88.08–99.45% identity between conspecific sequences and 29.92–79.98% between sequences assigned to different species. The two sets of figures derive from a single identity computation and differ only in whether the pairwise distribution is pooled across all sequence pairs or partitioned by taxonomic rank. This places P4 at a far higher level of conservation than the P3 glycoprotein-like product of the same viruses (≈13% mean identity; Section 3.5), as expected given the exceptional selective constraint on RdRp catalytic function [67]. The divergence is nonetheless substantial and consistent with family- rather than genus-level breadth. Pairwise identities are structured by host botanical family: sequences from viruses sharing a host family are appreciably more similar to one another (mean 50.5%) than to sequences from other host families (mean 38.8%), and the identity matrix resolves coherent within-family blocks, most conspicuously for *Orchidaceae*-, *Brassicaceae*- and *Balanophoraceae*-associated viruses (Supplementary Figure S1B).

#### 3.7.4. Conservation Is Concentrated in the RdRp Core, Mirroring the Structural Result

The per-site conservation profile provides a direct sequence-level counterpart to the structural observation (Supplementary Figure S1C). Conservation is not uniform along P4: the palmprint-bearing N-terminal moiety, corresponding to the structurally resolved RdRp core, is significantly more conserved than the C-terminal moiety that harbours the capping, methyltransferase and C-terminal domains (mean normalised conservation 0.69 versus 0.49; Mann–Whitney U test, one-sided, *p* ≈ 2 × 10^−65^). The three catalytic motifs sit within near-invariant columns (Shannon entropy ≈ 0.00–0.03 bits) against a genome-wide median column entropy of 1.92 bits, and 782 alignment columns are effectively invariant (entropy < 0.5 bits). Thus, the same partition seen structurally, a well-ordered, superimposable RdRp core versus poorly resolved accessory domains, is recovered as a quantitative divergence gradient in the primary sequence, with the accessory region evolving substantially faster than the core.

#### 3.7.5. The Catalytic Palmprint Is Invariant and Carries the Mononegavirales GDN Signature

At single-residue resolution, the catalytic apparatus is completely conserved across all 87 sequences (Supplementary Figure S1D–F). The metal-coordinating aspartate of motif A is present in 87/87 sequences within an invariant DFSKW block, and the catalytic triad of motif C is G-D-N in 87/87 sequences, embedded in the pentapeptide QGDNQ that is invariant in 86/87 sequences (the single exception, an A-for-G substitution at the first flanking position, leaves the catalytic GDN intact). Motif B (block GWEGQRQKGW) is conserved in 83/87 sequences (95.4%), with the four variants being conservative substitutions that preserve the block framework. No sequence carries a GDD or SDD variant at motif C.

The identity of the motif-C triad is phylogenetically informative. Among viral polymerases, the motif-C signature is group-specific: GDD in the RdRps of most positive-strand RNA viruses, SDD in the L proteins of segmented negative-strand RNA viruses, and GDN in the L proteins of the non-segmented negative-strand viruses of the *Mononegavirales* [68,69], where the GDNQ motif is functionally essential [70,71], and the QGDNQ pentapeptide is recognised as one of the longest invariant stretches of the polymerase domain [72]. The uniform GDN of P4 therefore places its polymerase squarely in the *Mononegavirales* catalytic class and is inconsistent with a segmented- or positive-strand origin. Together, the invariant motif-A aspartate and the invariant GDN of motif C reconstitute both catalytic aspartates of the two-metal-ion mechanism [64,68], providing strong sequence support for a catalytically competent polymerase.

The convergent structural and sequence evidence establishes P4 as a functional-grade, *Mononegavirales*-type RdRp, while also delimiting the confidence of that assignment domain by domain.

#### 3.7.6. The Catalytic Core Is Intact, and Its Assignment Is Robust

The recovery of a canonical fingers–palm–thumb fold that superimposes on VSV L (Figure 5E–G), combined with the 100% conservation of the motif-A aspartate and the motif-C GDN across all 87 viruses (Supplementary Figure S1D–F), makes the identification of P4 as an active RdRp about as secure as sequence and modelling evidence allow. The concordance is not trivial: structure alone can be over-interpreted at low homology, and sequence motifs alone can be recovered in pseudogenised or defective polymerases, but their agreement here, a resolved active-site geometry occupied by an invariant, correctly spaced catalytic constellation, is difficult to reconcile with anything other than a bona fide polymerase. The GDN signature further fixes the catalytic class as *Mononegavirales* rather than segmented (SDD) or positive-strand (GDD); consistent with this, experimental substitution of GDN by GDD or SDD abolishes or severely impairs L activity in rabies virus and VSV [68], so the uniform GDN of P4 is the signature expected of a functional non-segmented negative-strand polymerase and supports the family’s proposed placement as a plant-associated lineage within the *Mononegavirales*.

#### 3.7.7. The Unresolved Accessory Domains Most Plausibly Reflect Divergence, Not Absence

The failure of the capping, connector, methyltransferase, and C-terminal regions to superimpose on their VSV counterparts (Figure 5D–F) admits several explanations, and the sequence data help to discriminate among them. The first, that these domains are genuinely absent, is not supported: P4 is full-length and remarkably length-invariant (s.d. 18 aa over 87 sequences), leaving no room for large deletions of an entire capping or methyltransferase module. The second, that the region is intrinsically disordered, is also unlikely, because the C-terminal moiety retains appreciable sequence conservation (mean normalised conservation 0.49; Supplementary Figure S1C), well above what unconstrained sequence would show, indicating ongoing purifying selection rather than degeneration. The most parsimonious interpretation is the third: these are genuine but strongly divergent accessory domains whose remote homology to *Rhabdoviridae* fell below the confidence threshold of homology-based structural modelling, so that AlphaFold2 could neither template them on 5A22 nor place them by co-evolutionary signal. This interpretation is consistent with the pronounced core-versus-accessory conservation gradient (*p* ≈ 2 × 10^−65^), itself the expected signature of a conserved catalytic core carrying faster-evolving peripheral modules. We therefore describe the accessory domains as unresolved rather than lost, and caution against inferring their absence from the model.

Several caveats constrain the present claims. First, functional competence is inferred, not demonstrated; the catalytic assignment remains a strong hypothesis pending experimental evidence, which for these uncultured, metagenomically defined viruses will require heterologous expression and in vitro polymerase or minigenome assays. Second, we did not test the accessory region for the diagnostic sequence signatures of its putative functions, for example, the collinear PRNTase capping motifs A–E and the invariant HR of the capping active site [73,74], or the K-D-K-E tetrad of the 2′-O-methyltransferase [46]; confirming or refuting these would materially sharpen the interpretation of the unresolved domains and is a clear next step.

### 3.8. Genus-Level Classification and Host Associations

Based on phylogenetic analysis of RdRp sequences and pairwise identity thresholds, we propose the classification of *Masuviridae* into 15 genera designated *Alphamasuvirus* through *Omicronmasuvirus* following established nomenclature conventions (Figure 1C,D). This taxonomic framework is supported by both molecular phylogeny and distinct ecological patterns in host plant associations.

The genus assignments were established using a combination of phylogenetic clustering and RdRp amino acid identity thresholds, with genera showing discrete monophyletic groups and identity values typically below 40% between genera. Within genera, virus species exhibited higher sequence conservation (>40% identity) while maintaining distinct host ecological niches. The largest genus, *Zetamasuvirus*, encompasses 19 virus species, while several genera (*Gammamasuvirus*, *Iotamasuvirus*, *Lambdamasuvirus*) are currently represented by single species, reflecting either restricted geographic sampling or genuine ecological specialization.

Analysis of 89 confirmed virus–host associations across the 15 genera revealed striking patterns of host specificity that support the proposed taxonomic divisions. Most notably, several genera exhibit strong host plant family specialization. *Numasuvirus*, *Kappamasuvirus*, and *Lambdamasuvirus* are exclusively associated with *Orchidaceae*, with *Numasuvirus* showing the broadest orchid host range (6 viruses across 4 orchid species). Similarly, *Betamasuvirus* demonstrates complete fidelity to *Brassicaceae* (8 viruses), while *Alphamasuvirus* shows strong preference for this family (71% of associations). The most extreme case of host restriction occurs in *Epsilonmasuvirus*, where all seven virus isolates derive from a single host species, *Rhopalocnemis phalloides* (Balanophoraceae), suggesting either recent host colonization or highly specialized adaptation.

In contrast, *Zetamasuvirus* and *Mumasuvirus* exhibit broader host ranges spanning 9 and 6 plant families, respectively, with *Asteraceae* representing the most frequent host family overall (24 virus associations). This pattern suggests these genera may represent either ancestral generalist lineages or taxa with enhanced host-switching capabilities.

The observed host associations likely reflect multiple evolutionary and ecological factors, including geographic sampling bias toward agricultural species, phylogenetic constraints on host compatibility, and vector specificity where applicable. The predominance of dicot hosts (78% of associations) and the concentration of viruses in economically important plant families (*Asteraceae*, *Brassicaceae*, *Orchidaceae*) may partly reflect detection bias in transcriptomic datasets. However, the genus-specific clustering of host associations indicates genuine biological constraints rather than purely technical artifacts.

These host association patterns provide additional support for the proposed genus-level classification and suggest that ecological specialization has been a significant driver in *Masuviridae* diversification. The combination of phylogenetic divergence and host specificity patterns observed here parallels those documented in other plant virus families where host adaptation and geographic isolation promote speciation and taxonomic diversification.

### 3.9. Viral Diversity and Prevalence Patterns

We employed the Logan search tool (https://logan-search.org/dashboard, accessed on 30 July 2026) [41] to explore all publicly available libraries in order to search for the newly identified lispi-like virus sequences as well as MSuV and Disporopsis pernyi associated lispi-like viruses. Our comprehensive data mining identified 1536 public RNA-seq libraries containing lispi-like viral sequences across 134 host plant species, representing 11 plant families from 182 geographic locations worldwide. This substantial viral diversity, accumulated over nine years of sequencing data (2015–2024), demonstrates the power of retrospective bioinformatic mining to uncover previously unrecognized plant virus diversity hiding within transcriptomic datasets generated for other research purposes. Furthermore, it is tempting to speculate that substantial remaining diversity is still hidden; thus, a comprehensive sampling of global plant diversity could reveal hundreds of additional lispi-like viruses.

The application of a normalized Virus Prevalence Score (VPS), calculated as (Unique Hosts/√Libraries) × 100, is key to understanding the virus importance compared to raw detection frequencies (Supplementary Figures S2 and S3). While Brassica lispi-like virus 2 dominated the raw library counts with 509 detections, VPS normalization revealed Astragalus lispi-like virus 1 as the most ecologically prevalent (VPS = 163), infecting diverse hosts despite fewer absolute detections. This normalization discounts, without fully cancelling, the sampling bias inherent in heavily studied agricultural systems and model organisms, providing a sampling-adjusted ranking of host-range breadth rather than an absolute measure of it. Because the denominator is the square root of the library count, VPS is not a proportion and is not bounded at 100 (Section 2.15), and it is therefore interpreted here only alongside the raw library and unique-host counts from which it derives. This disparity between raw counts and normalized prevalence underscores that detection frequency often reflects research intensity rather than biological significance [75], a critical consideration for data-mining studies that must contend with highly uneven sampling across the plant kingdom.

The relationship between library abundance and host diversity revealed distinct ecological strategies among the identified viruses (Supplementary Figure S4). Brassica-lispi-like viruses 2 and 3 exemplify specialists with narrow host ranges but high detection frequencies within their preferred hosts (18–20 organisms, primarily *Brassicaceae*). In contrast, viruses like the Astragalus lispi-like virus 1, despite moderate library counts, demonstrated broader host diversity with high detection robustness, suggesting true generalist capabilities rather than sampling artifacts. Raphanus lispi-like virus 1 occupied an intermediate position with both substantial library counts and moderate host diversity (14 organisms), potentially representing an emerging generalist lineage. This diversity of ecological strategies parallels patterns observed in other plant virus families, where host range evolution proceeds along multiple trajectories shaped by vector specificity and cellular compatibility, as well as geographic opportunity [76,77].

### 3.10. Host Specificity and Range Constraints

Analysis of host specificity patterns revealed that strict host specialization predominates among lispi-like viruses, with 53 of 75 viruses (70.7%) restricted to single host species. This extreme specialization contrasts with the small number of generalists capable of infecting 4–20 different hosts (n = 6 viruses, 8%). The negative correlation between sampling intensity and host specificity index suggests that apparent generalism may partially reflect detection bias, where those viruses sampled more intensively have greater opportunity to reveal additional hosts. However, the existence of true specialists even among heavily sampled viruses (e.g., Brassica lispi-like virus 3 with 172 libraries but restricted to *Brassicaceae*) indicates that biological constraints, not merely sampling limitations, define host range boundaries for the lispi-like viruses, such as has been reported for other plant viruses [77,78].

Moreover, the comparison of monocot-specific versus dicot-dominated viruses revealed no significant difference in host diversity, where Dicot-dominated viruses (green, n = 109) show a broader distribution with a median around 1–2 hosts and outliers extending to 20 hosts, whereas Monocot-specific viruses (coral, n = 25) display a narrower distribution centered around 1–2 hosts with a maximum diversity of 5 hosts (Supplementary Figure S5). However, the Wilcoxon test (*p* = 0.3636) (Supplementary Figure S5) indicates no statistically significant difference in host diversity between groups, suggesting that host range breadth is not determined by the monocot/dicot classification of primary hosts.

### 3.11. Library Redundancy and Sampling Bias

The substantial discrepancy between total library counts and unique BioProjects for top-ranking viruses highlights a critical challenge in interpreting data-mining results (Supplementary Figure S6). Brassica lispi-like virus 2, with 509 libraries derived from only 72 independent studies, demonstrates how intensive sampling of specific experimental systems can inflate apparent prevalence. This redundancy particularly affects agricultural and model organism systems where researchers conduct time-series experiments, biological replicates, and multi-condition comparisons, all generating separate SRA entries that represent the same geographic population or experimental lineage [79].

Tissue tropism analysis revealed strong bias toward foliar sampling, with leaf tissue representing >75% of detections for most viruses (Supplementary Figure S6). This sampling artifact limits our ability to assess true tissue specificity, where viruses appearing leaf-specific may simply reflect that leaves are the predominantly sampled tissue [80]. The exceptions, such as Raphanus lispi-like virus 1 and Brassica lispi-like virus 3 with documented flower, root, stem, and whole plant detections, suggest these viruses either possess genuine systemic infection capability or have been subjects of more comprehensive tissue surveys. Seed-associated detections for Ageratum-lispi-like viruses 1 and 2 and Arnica lispi-like virus 1, as well as flower-associated detections for Monotropastrum lispi-like virus 1, Ophrys lispi like virus 1, Caladenia lispi like virus 1 and Gymnadenia lispi-like virus 1 (Supplementary Figure S6) raise questions about vertical transmission potential that cannot be addressed without targeted experimental follow-up. Other negative-sense plant viruses were reported to be seed-associated [81,82,83], and the lispi-like virus MSuV was found to be seed-transmitted [28], supporting the hypothesis that lispi-like viruses could be vertically transmitted.

### 3.12. Agricultural Relevance and Wild Plant Reservoirs

The agricultural relevance analysis revealed a dichotomy between crop-associated viruses and those circulating in wild plant populations. Brassica lispi-like viruses 1 and 2 showed >95% association with major crops despite limited host diversity, representing viruses of immediate agricultural concern that may cause unreported or subclinical impacts on crop productivity. In contrast, numerous lispi-like viruses detected exclusively in wild plants represent a largely unexplored reservoir of viral diversity with unknown agricultural risk. These wild plant viruses could potentially represent preadapted lineages capable of emerging into agricultural systems, similar to historical examples where crop domestication or agricultural intensification facilitated virus host shifts [75]. Raphanus lispi-like virus 1, with ~75% crop association across 14 hosts, may exemplify an intermediate stage where viruses maintain circulation in both agricultural and wild populations, potentially using wild hosts as reservoirs for recolonization after crop rotations or seasonal cycles.

### 3.13. Geographic Distribution

Global sampling coverage (Supplementary Figure S7) revealed strong geographic bias toward northern hemisphere temperate regions, particularly East Asia, Europe, and North America. China emerged as a hotspot with the highest virus richness (more than 15 viruses), likely reflecting both genuine biodiversity and substantial investment in plant genomics research. While sampling extended to 182 unique locations, density remained highest in regions with established agricultural research infrastructure and genomics capacity. This geographic bias has important implications for understanding global viral biogeography. Tropical and southern hemisphere regions remain undersampled despite harboring the majority of plant species diversity [84,85], suggesting substantial undiscovered viral diversity in these areas.

**Table 1:** Summary of novel plant-associated lispi-like viruses identified from plant RNA-seq data available on NCBI.

Plant Host	Taxa/ Family	Virus Name/ Abbreviation	Bioproject ID/ Data Citation	Length (nt)/ Coverage	Accession Number	Protein ID	Length (aa)	Highest Scoring Virus-Protein/*E*-Value/Query Coverage%/Identity% (BLASTP)
floss flower (*Ageratum houstonianum*)	Dicot/ *Asteraceae*	Ageratum lispi-like virus 1/ AgeLlV1	PRJNA371565/ [86]	13020/226.31X	BK075274	N P2 P3 L	397 221 616 2359	MSV-N/6 × 10^−22^/52/31.1 no hits no hits MSV-L/0.0/100/34.31
floss flower (*Ageratum houstonianum*)	Dicot/ *Asteraceae*	Ageratum lispi-like virus 2/ AgeLlV2	PRJNA371565/ [86]	12086/218.45X	BK075275	N P2 P3 L	384 236 644 2382	MSV-N/3 × 10^−32^/78/28.57 no hits no hits MSV-L/0.0/100/44.22
Welsh onion (*Allium fistulosum*)	Monocot/ *Allieae*	Allium lispi-like virus 1/ AllLlV1	PRJNA542932/ [87]	12047/44.05X	BK075276	N P2 P3 L	379 218 676 2341	MSV-N/4 × 10^−56^/96/32.6 no hits no hits MSV-L/0.0/99/45.91
wild garlic (*Allium ursinum*)	Monocot/ *Allieae*	Allium lispi-like virus 2/ AllLlV2	PRJNA542932/ [87]	13318/421.95X	BK075277	N P2 P3 L	417 271 695 2382	MSV-N/8 × 10^−21^/64/28.1 no hits no hits MSV-L/0.0/98/43.88
alpine leek (*Allium victoralis*)	Monocot/ *Allieae*	Allium lispi-like virus 3/ AllLlV3	PRJNA633672/ [88]	11800/163.99X	BK075278	N P2 P3 L	363 219 627 2341	MSV-N/8 × 10^−52^/88/31.37 no hits no hits MSV-L/0.0/100/45.44
common ragweed (*Ambrosia artemisiifolia*)	Dicot/ *Asteraceae*	Ambrosia lispi-like virus 1/ AmbLlV1	PRJNA335689/ [89]	12316/12.49X	BK075279	N P2 P3 L	381 225 619 2374	MSV-N/7 × 10^−39^/71/34.8 no hits no hits MSV-L/0.0/100/41.85
sand rock-cress (*Arabidopsis arenosa*)	Dicot/ *Brassicaceae*	Arabidopsis lispi-like virus 1/ AraLlV1	PRJNA575330/ [90]	12590/23.01X	BK075280	N P2 P3 L	383 243 614 2339	MSV-N/2 × 10^−29^/74/28.62 no hits no hits MSV-L/0.0/99/40.33
dwarf mistletoe (*Arceuthobium sichuanense*)	Dicot/ *Santalaceae*	Arceuthobium lispi-like virus 1/ ArcLlV1	PRJNA307530/ [91]	12177/69.03X	BK075281	N P2 P3 L	359 247 695 2353	MSV-N/1 × 10^−24^/74/31.7 no hits no hits MSV-L/0.0/100/40.19
mountain tobacco (*Arnica montana*)	Dicot/ *Asteraceae*	Arnica lispi-like virus 1/ ArnLlV1	PRJNA233448/ [92]	11732/23.84X	BK075282	N P2 P3 L	367 220 655 2338	MSV-N/7 × 10^−40^/84/31.09 no hits no hits MSV-L/0.0/100/45.26
Chinese milkvetch (*Astragalus sinicus*)	Dicot/ *Fabaceae*	Astragalus lispi-like virus 1/ AstLlV1	PRJNA690069/ [93]	13281/29.38X	BK075283	N P2 P3 L	370 239 669 2343	MSV-N/2 × 10^−16^/77/25.5 no hits no hits MSV-L/0.0/100/36.11
Bai Zhu (*Atractylodes macrocephala*)	Dicot/ *Asteraceae*	Atractylodes lispi-like virus 1/ AtrLlV1	PRJNA419787/ [94]	12540/56.48X	BK075284	N P2 P3 L	406 211 636 2338	MSV-N/2 × 10^−27^/66/27.61 no hits no hits MSV-L/0.0/100/35.74
black mustard (*Brassica nigra*)	Dicot/ *Brassicaceae*	Brassica lispi-like virus 1/ BraLlV1	PRJNA309775/ He, Z., York University, unpublished	12516/16.43X	BK075285	N P2 P3 L	405 244 564 2345	MSV-N/9 × 10^−33^/71/28.47 no hits no hits MSV-L/0.0/100/38.38
rapeseed (*Brassica napus*)	Dicot/ *Brassicaceae*	Brassica lispi-like virus 2_nap/ BraLlV2_nap	PRJNA549345/ [95]	12489/38.49X	BK075286	N P2 P3 L	402 244 573 2385	MSV-N/5 × 10^−22^/73/24.83 no hits no hits MSV-L/0.0/99/38.4
turnip (*Brassica rapa*)	Dicot/ *Brassicaceae*	Brassica lispi-like virus 2_rap/ BraLlV2_rap	PRJNA312975/ Zhong, L., SCAU, China, unpublished	12253/15.22X	BK075287	N P2 P3 L	402 244 573 2385	MSV-N/5 × 10^−22^/73/24.83 no hits no hits MSV-L/0.0/99/38.4
short-pod mustard (*Brassica Incana*)	Dicot/ *Brassicaceae*	Brassica lispi-like virus 3_inc/ BraLlV3_inc	PRJNA428769/ [96]	12222/23.94X	BK075288	N P2 P3 L	387 232 621 2341	MSV-N/2 × 10^−34^/87/30.68 no hits no hits MSV-L/0.0/99/38.78
Indian mustard (*Brassica insularis*)	Dicot/ *Brassicaceae*	Brassica lispi-like virus 3_ins/ BraLlV3_ins	PRJNA428769/ [96]	12088/15.16X	BK075289	N P2 P3 L	387 232 622 2341	MSV-N/2 × 10^−34^/87/30.68 no hits no hits MSV-L/0.0/99/38.78
wild cabbage (*Brassica oleracea*)	Dicot/ *Brassicaceae*	Brassica lispi-like virus 3_ole/ BraLlV3_ole	PRJNA428769/ [96]	12183/42.49X	BK075290	N P2 P3 L	387 232 621 2341	MSV-N/2 × 10^−34^/87/30.68 no hits no hits MSV-L/0.0/99/38.78
mustard (*Brassica rupestris*)	Dicot/ *Brassicaceae*	Brassica lispi-like virus 3_rup/ BraLlV3_rup	PRJNA428769/ [96]	12230/14.47X	BK075291	N P2 P3 L	387 232 621 2341	MSV-N/2 × 10^−34^/87/30.68 no hits no hits MSV-L/0.0/99/38.78
Sicilian wild cabbage (*Brassica villosa*)	Dicot/ *Brassicaceae*	Brassica lispi-like virus 3_vil/ BraLlV3_vil	PRJNA428769/ [96]	12141/22.14X	BK075292	N P2 P3 L	387 232 621 2341	MSV-N/2 × 10^−34^/87/30.68 no hits no hits MSV-L/0.0/99/38.78
mantis orchid (*Caladenia attigens*)	Monocot/ *Orchidaceae*	Caladenia lispi-like virus 1_att/ CalLlV1_att	PRJNA661963/ [97]	11452/27.89X	BK075293	N P2 P3 L	386 225 549 2324	MSV-N/2 × 10^−34^/78/30.59 no hits no hits MSV-L/0.0/100/40.11
crab-lipped spider orchid (*Caladenia plicata*)	Monocot/ *Orchidaceae*	Caladenia lispi-like virus 1_pli/ CalLlV1_pli	PRJNA384875/ [98]	11404/21.43X	BK075294	N P2 P3 L	386 225 549 2324	MSV-N/2 × 10^−34^/78/30.59 no hits no hits MSV-L/0.0/100/40.11
Canberra spider orchid (*Caladenia actensis*)	Monocot/ *Orchidaceae*	Caladenia lispi-like virus 2_act/ CalLlV2_act	PRJNA661963/ [97]	11193/107.06X	BK075295	N P2 P3 L	394 217 489 2314	MSV-N/5 × 10^−37^/76/32.57 no hits no hits MSV-L/0.0/100/39.86
Arrowsmith spider orchid (*Caladenia crebra*)	Monocot/ *Orchidaceae*	Caladenia lispi-like virus 2_cre/ CalLlV2_cre	PRJNA661963/ [97]	11499/73.94X	BK075296	N P2 P3 L	391 217 489 2314	MSV-N/4 × 10^−33^/74/32.23 no hits no hits MSV-L/0.0/100/39.86
hemp (*Cannabis sativa*)	Dicot/ *Cannabaceae*	Cannabis lispi-like virus 1/ CanLlV1	PRJNA773629/ Yue, J., CAMS, China, unpublished	13200/580.65X	BK075297	N P2 P3 L	382 224 714 2344	MSV-N/1 × 10^−38^/80/32.79 no hits no hits MSV-L/0.0/100/42.93
grand Shepherd’s-purse (*Capsella grandiflora*)	Dicot/ *Brassicaceae*	Capsella lispi-like virus 1/ CapLlV1	PRJNA533007/ [99]	12782/63.95X	BK075298	N P2 P3 L	381 235 573 2344	MSV-N/2 × 10^−22^/90/26.42 no hits no hits MSV-L/0.0/100/39.68
carrot (*Daucus carota*)	Dicot/ Apiaceae	carrot lispi-like virus 1/ CarLlV1	PRJNA574412/ [100]	13434/524.80X	BK075299	N P2 P3 L	458 220 644 2320	MSV-N/4 × 10^−24^/61/27.92 no hits no hits MSV-L/0.0/100/33.42
pearl millet (*Cenchrus americanus*)	Monocot/ *Poaceae*	Cenchrus lispi-like virus 1/ CenLlV1	PRJNA625418/ Kumar, R., ICAR, India, unpublished	12133/352.96X	BK075300	N P2 P3 L	369 226 675 2328	MSV-N/2 × 10^−38^/91/27.84 no hits no hits MSV-L/0.0/100/44.8
cornflower (*Centaurea cyanus*)	Dicot/ *Asteraceae*	Centaurea lispi-like virus 1/ CentLlV1	PRJNA577252/ [101]	12996/12.07X	BK075301	N P2 P3 L	393 215 636 2341	MSV-N/4 × 10^−29^/69/28.47 no hits no hits MSV-L/0.0/100/36.56
common chicory (*Cichorium intybus*)	Dicot/ *Asteraceae*	Cichorium lispi-like virus 1/ CicLlV1	PRJNA328202/ [102]	12530/18.57X	BK075302	N P2 P3 L	389 217 644 2335	MSV-N/8 × 10^−18^/55/29.77 no hits no hits MSV-L/0.0/99/36.18
desert ginseng (*Cistanche deserticola*)	Dicot/ *Orobanchaceae*	Cistanche lispi-like virus 1/ CisLlV1	PRJNA598927/ Li, M., Gansu, China, unpublished	12415/237.02X	BK075303	N P2 P3 L	396 245 659 2344	MSV-N/3 × 10^−34^/78/29.52 no hits no hits MSV-L/0.0/100/39-29
desert ginseng (*Cistanche deserticola*)	Dicot/ *Orobanchaceae*	Cistanche lispi-like virus 2/ CisLlV2	PRJNA598927/ Li, M., Gansu, China, unpublished	12552/112.82X	BK075304	N P2 P3 L	396 245 659 2344	MSV-N/5 × 10^−28^/59/33.77 no hits no hits MSV-L/0.0/100/40.73
golden thread (*Coptis chinensis*)	Dicot/ *Ranunculaceae*	Coptis lispi-like virus 1/ CopLlV1	PRJNA662860/ [103]	12062/114.68X	BK075305	N P2 P3 L	391 236 598 2342	MSV-N/2 × 10^−46^/86/32.25 no hits no hits MSV-L/0.0/100/40.3
coriander (*Coriandrum sativum*)	Dicot/ Apiaceae	Coriandrum lispi-like virus 1/ CorLlV1	PRJNA531360/ [104]	12694/14.25X	BK075306	N P2 P3 L	431 215 691 2346	MSV-N/1 × 10^−25^/62/29.93 no hits no hits MSV-L/0.0/100/34.84
coriander (*Coriandrum sativum*)	Dicot/ Apiaceae	Coriandrum lispi-like virus 2/ CorLlV2	PRJNA472685/ [105]	12365/12.79X	BK075307	N P2 P3 L	408 238 654 2343	MSV-N/2 × 10^−38^/86/33.25 no hits no hits MSV-L/0.0/100/44.68
desert dodder (*Cuscuta nevadensis*)	Dicot/ *Convolvulaceae*	Cuscuta lispi-like virus 1/ CusLlV1	PRJNA561399/ Frangione E, U Toronto, Canada, unpublished	12694/41.36X	BK075308	N P2 P3 L	416 224 701 2329	MSV-N/2 × 10^−29^/65/29.89 no hits no hits MSV-L/0.0/100/35.25
Taiwan spoon orchid (*Cypripedium formosanum*)	Monocot/ *Orchidaceae*	Cypripedium lispi-like virus 1/ CypLlV1	PRJNA277578/ [106]	12935/71.96X	BK075309	N P2 P3 L	369 226 684 2357	MSV-N/3 × 10^−45^/93/31.52 no hits no hits MSV-L/0.0/100/43.09
Yellow slipper orchid (*Cypripedium flavum*)	Monocot/ *Orchidaceae*	Cypripedium lispi-like virus 2/ CypLlV2	PRJNA479379/ [107]	12546/85.93X	BK075310	N P2 P3 L	381 223 694 2342	MSV-N/3 × 10^−34^/76/30.03 no hits no hits MSV-L/0.0/100/39.95
common spotted-orchid (*Dactylorhiza fuchsii*)	Monocot/ *Orchidaceae*	Dactylorhiza lispi-like virus 1/ DacLlV1	PRJNA317244/ [108]	11802/18.81X	BK075311	N P2 P3 L	368 223 628 2353	MSV-N/2 × 10^−32^/75/31.32 no hits no hits MSV-L/0.0/100/37.28
common spotted-orchid (*Dactylorhiza fuchsii*)	Monocot/ *Orchidaceae*	Dactylorhiza lispi-like virus 2/ DacLlV2	PRJNA317244/ [108]	12298/27.35X	BK075312	N P2 P3 L	376 232 612 2402	MSV-N/2 × 10^−36^/79/31.79 no hits no hits MSV-L/0.0/100/35.05
common spotted-orchid (*Dactylorhiza fuchsii*)	Monocot/ *Orchidaceae*	Dactylorhiza lispi-like virus 3/ DacLlV3	PRJNA317244/ [108]	11960/50.36X	BK075313	N P2 P3 L	367 222 620 2353	MSV-N/6 × 10^−32^/77/31.27 no hits no hits MSV-L/0.0/100/37.33
Venus flytrap (*Dionaea muscipula*)	Dicot/ Droseraceae	Dionaea lispi-like virus 1/ DioLlV1	PRJNA203407/ [109]	12600/266.77X	BK075314	N P2 P3 L	369 236 658 2344	MSV-N/2 × 10^−43^/75/34.04 no hits no hits MSV-L/0.0/100/41.73
violet helleborine (*Epipactis purpurata*)	Monocot/ *Orchidaceae*	Epipactis lispi-like virus 1/ EpiLlV1	PRJNA450088/ [110]	13620/169.66X	BK075315	N P2 P3 L	374 219 651 2345	MSV-N/1 × 10^−46^/79/34.01 no hits no hits MSV-L/0.0/100/41.05
violet helleborine (*Epipactis purpurata*)	Monocot/ *Orchidaceae*	Epipactis lispi-like virus 2/ EpiLlV2	PRJNA450088/ [110]	12946/120.39X	BK075316	N P2 P3 L	391 219 678 2349	MSV-N/4 × 10^−37^/85/29.55 no hits no hits MSV-L/0.0/100/40.21
shortscape fleabane (*Erigeron breviscapus*)	Dicot/ *Asteraceae*	Erigeron lispi-like virus 1/ EriLlV1	PRJNA293262/ [111]	11755/33.58X	BK075317	N P2 P3 L	375 222 649 2338	MSV-N3e-43/95/31.97 no hits no hits MSV-L/0.0/100/45.88
garden rocket (*Eruca vesicaria*)	Dicot/ *Brassicaceae*	Eruca lispi-like virus 1_eru/ EruLlV1_eru	PRJNA475309/ [112]	12152/53.71X	BK075318	N P2 P3 L	401 243 569 2342	MSV-N/2 × 10^−23^/72/26.71 no hits no hits MSV-L/0.0/99/38.5
wallflower (*Erysimum cheiri*)	Dicot/ *Brassicaceae*	Eruca lispi-like virus 1_ery/ EruLlV1_ery	PRJNA362874/ [113]	12427/73.70X	BK075319	N P2 P3 L	401 243 569 2395	MSV-N/2 × 10^−23^/72/26.71 no hits no hits MSV-L/0.0/99/37.72
Bastetan wallflower (*Erysimum bastetanum*)	Dicot/ *Brassicaceae*	Erysimum lispi-like virus 1/ EryLlV1	PRJNA607615/ [114]	12670/275.88X	BK075320	N P2 P3 L	411 235 587 2389	MSV-N/6 × 10^−19^/74/25.9 no hits no hits MSV-L/0.0/98/37.17
niger (*Guizotia abyssinica*)	Dicot/ *Asteraceae*	Guizotia lispi-like virus 1/ GuiLlV1	PRJNA763316/ [115]	12071/23.56X	BK075321	N P2 P3 L	377 221 717 2336	MSV-N/2 × 10^−42^/88/30.27 no hits no hits MSV-L/0.0/100/45.42
marsh-fragrant orchid (*Gymnadenia densiflora*)	Monocot/ *Orchidaceae*	Gymnadenia lispi-like virus 1/ GymLlV1	PRJNA504609/ [116]	12203/145.36X	BK075322	N P2 P3 L	367 222 634 2355	MSV-N/1 × 10^−32^/76/31.01 no hits no hits MSV-L///
very small catchfly (*Heliosperma pusillum*)	Dicot/ *Caryophyllaceae*	Heliosperma lispi-like virus 1/ HelLlV1	PRJNA760819/ [117]	12884/271.81X	BK075323	N P2 P3 L	396 233 632 2362	MSV-N/6 × 10^−21^/85/26.53 no hits no hits MSV-L/0.0/99/36.89
wild hogweed (*Heracleum moellendorffii*)	Dicot/ *Apiaceae*	Heracleum lispi-like virus 1/ HerLlV1	PRJNA438242 [118]	12352/16.98X	BK075324	N P2 P3 L	402 237 654 2342	MSV-N/9 × 10^−31^/88/27.81 no hits no hits MSV-L/0.0/100/44.28
lily (*Ixiolirion sp*.)	Monocot/ *Ixioliriaceae*	Ixiolirion lispiu-like virus 1/ IxiLlV1	PRJNA412930/ [119]	11807/80.61X	BK075325	N P2 P3 L	385 247 583 2341	MSV-N/4 × 10^−29^/90/29.05 no hits no hits MSV-L/0.0/99/39.92
Indian lettuce (*Lactuca indica*)	Dicot/ *Asteraceae*	Lactuca lispi-like virus 1/ LacLlV1	PRJNA386610/ [120]	12952/52.91X	BK075326	N P2 P3 L	388 223 632 2345	MSV-N/2 × 10^−24^/85/24.77 no hits no hits MSV-L/0.0/98/39.84
sea lavender (*Limonium bicolor*)	Dicot/ *Plumbaginaceae*	Limonium lispi-like virus 1/ LimLlV1	PRJNA752802/ [121]	12676/14.84X	BK075327	N P2 P3 L	377 220 630 2340	MSV-N/1 × 10^−31^/78/31.65 no hits no hits MSV-L/0.0/100/38.93
anual mercuri (*Merculiaris annua*)	Dicot/ *Euphorbiaceae*	Mercurialis lispi-like virus 1/ MerLlV1	PRJNA369310/ [122]	13024/174.77X	BK075328	N P2 P3 L	412 236 615 2325	MSV-N/7 × 10^−20^/62/29.17 no hits no hits MSV-L/0.0/98/37.02
ghost plant (*Monotropastrum humile*)	Dicot/ *Ericaceae*	Monotropastrum lispi-like virus 1/ MonLlV1	PRJDB3033/ NIBB, Japan, unpublished	12029/101.32X	BK075329	N P2 P3 L	375 227 632 2347	MSV-N/4 × 10^−35^/74/33.09 no hits no hits MSV-L/0.0/100/40.92
violet cabbage (*Moricandia arvensis*)	Dicot/ *Brassicaceae*	Moricandia lispi-like virus 1/ MorLlV1	PRJNA604514/ [123]	12409/228.54X	BK075330	N P2 P3 L	402 244 571 2346	MSV-N/4 × 10^−21^/69/24.55 no hits no hits MSV-L/0.0/98/38.19
dark bee orchid (*Ophrys fusca*)	Monocot/ *Orchidaceae*	Ophrys lispi-like virus 1/ OphLlV1	PRJNA574279 [116]	12020/15.87X	BK075331	N P2 P3 L	371 231 637 2353	MSV-N/4 × 10^−31^/90/29.13 no hits no hits MSV-L/0.0/100/37.95
early spider orchid (*Ophrys sphegodes*)	Monocot/ *Orchidaceae*	Ophrys lispi-like virus 2/ OphLlV2	PRJNA574279 [116]	11574/34.56X	BK075332	N P2 P3 L	371 223 638 2353	MSV-N/2 × 10^−32^/80/29.34 no hits no hits MSV-L/0.0/100/37.45
Chinese violet cree (*Orychophragmus violaceus*)	Dicot/ *Brassicaceae*	Orychophragmus lispi-like virus 1/ OryLlV1	PRJNA431031/ Zhong, L., Sichuan U, China, unpublished	12197/39.46X	BK075333	N P2 P3 L	390 231 597 2340	MSV-N/4 × 10^−28^/84/28.57 no hits no hits MSV-L/0.0/93/41.09
herb Paris (*Paris polyphylla*)	Monocot/ *Melanthiaceae*	Paris lispi-like virus 1/ ParLlV1	PRJNA783190/ [124]	12001/85.56X	BK075334	N P2 P3 L	390 226 660 2348	MSV-N/2 × 10^−25^/76/29.57 no hits no hits MSV-L/0.0/99/36.16
desertbells (*Phacelia campanularia*)	Dicot/ *Boraginaceae*	Phacelia lispi-like virus 1/ PhaLlV1	PRJEB21674/ OneKP project, unpublished	12932/22.04X	BK075335	N P2 P3 L	374 234 618 2343	MSV-N/1 × 10^−30^/75/29.79 no hits no hits MSV-L/0.0/100/36.25
ribwort plantain (*Plantago lanceolata*)	Dicot/ *Plantaginaceae*	Plantago lispi-like virus 1/ PlaLlV1	PRJNA636383/ [125]	12367/32.36X	BK075336	N P2 P3 L	465 223 587 2340	MSV-N/5 × 10^−13^/44/29.14 no hits no hits MSV-L/0.0/99/35.68
garden radish (*Raphanus sativus*)	Dicot/ *Brassicaceae*	Raphanus lispi-like virus 1/ RapLlV1	PRJNA650223/ [126]	12500/515.06X	BK075337	N P2 P3 L	388 239 597 2340	MSV-N/1 × 10^−18^/88/24.93 no hits no hits MSV-L/0.0/99/39.37
stinkhorn clubhead (*Rhopalocnemis phalloides*)	Dicot/ *Balanophoraceae*	Rhopalocnemis lispi-like virus 1/ RhoLlV1	PRJNA737177/ [127]	11830/129.19X	BK075338	N P2 P3 L	396 259 561 2346	MSV-N/5 × 10^−22^/56/30.97 no hits no hits MSV-L/0.0/100/37.83
stinkhorn clubhead (*Rhopalocnemis phalloides*)	Dicot/ *Balanophoraceae*	Rhopalocnemis lispi-like virus 2/ RhoLlV2	PRJNA737177/ [127]	12460/183.34X	BK075339	N P2 P3 L	381 254 568 2343	MSV-N/1 × 10^−37^/87/30.54 no hits no hits MSV-L/0.0/100/38.65
stinkhorn clubhead (*Rhopalocnemis phalloides*)	Dicot/ *Balanophoraceae*	Rhopalocnemis lispi-like virus 3/ RhoLlV3	PRJNA737177/ [127]	11760/132.53X	BK075340	N P2 P3 L	395 234 545 2344	MSV-N/1 × 10^−36^/70/31.05 no hits no hits MSV-L/0.0/100/38.26
stinkhorn clubhead (*Rhopalocnemis phalloides*)	Dicot/ *Balanophoraceae*	Rhopalocnemis lispi-like virus 4/ RhoLlV4	PRJNA495456/ [128]	12177/280.79X	BK075341	N P2 P3 L	401 240 560 2340	MSV-N/7 × 10^−37^/81/31.63 no hits no hits MSV-L/0.0/100/37.76
stinkhorn clubhead (*Rhopalocnemis phalloides*)	Dicot/ *Balanophoraceae*	Rhopalocnemis lispi-like virus 5/ RhoLlV5	PRJNA495456/ [128]	12030/681.54X	BK075342	N P2 P3 L	401 234 546 2344	MSV-N/1 × 10^−29^/52/32.31 no hits no hits MSV-L/0.0/100/38.46
stinkhorn clubhead (*Rhopalocnemis phalloides*)	Dicot/ *Balanophoraceae*	Rhopalocnemis lispi-like virus 6/ RhoLlV6	PRJNA495456/ [128]	11897/909.17X	BK075343	N P2 P3 L	405 234 546 2344	MSV-N/1 × 10^−29^/51/33.97 no hits no hits MSV-L/0.0/100/38.08
stinkhorn clubhead (*Rhopalocnemis phalloides*)	Dicot/ *Balanophoraceae*	Rhopalocnemis lispi-like virus 7/ RhoLlV7	PRJNA495456/ [128]	11955/561.56X	BK075344	N P2 P3 L	387 250 571 2345	MSV-N/3 × 10^−26^/72/27.96 no hits no hits MSV-L/0.0/100/37.92
heartwing dock (*Rumex hastatulus*)	Dicot/ *Polygonaceae*	Rumex lispi-like virus 1/ RumLlV1	PRJNA472058/ [129]	12245/277.42X	BK075345	N P2 P3 L	378 223 652 2340	MSV-N/9 × 10^−50^/76/36.68 no hits no hits MSV-L/0.0/100/45.74
alpine sheep sorrell (*Rumex paucifolius*)	Dicot/ *Polygonaceae*	Rumex lispi-like virus 2/ RumLlV2	PRJNA698922/ [130]	11965/91.58X	BK075346	N P2 P3 L	378 222 656 2340	MSV-N/5 × 10^−45^/76/34.59 no hits no hits MSV-L/0.0/100/45.4
Chinese laserwort (*Saposhnikovia divaricata*)	Dicot/ *Apiaceae*	Saposhnikovia lispi-like virus 1/ SapLlV1	PRJNA613937/ [131]	13007/12.98X	BK075347	N P2 P3 L	467 220 665 2392	MSV-N/4 × 10^−26^/59/29.35 no hits no hits MSV-L/0.0/99/34.33
pincushion flower (*Scabiosa columbaria*)	Dicot/ *Caprifoliaceae*	Scabiosa lispi-like virus 1/ ScaLlV1	PRJNA80113/ [132]	12732/82.29X	BK075348	N P2 P3 L	403 228 696 2345	MSV-N/9 × 10^−22^/87/23.51 no hits no hits MSV-L/0.0/100/43.17
white campion (*Silene latifolia*)	Dicot/ *Caryophyllaceae*	Silene lispi-like virus 1_lat/ SilLlV1_lat	PRJEB39526/ [133]	12359/114.54X	BK075349	N P2 P3 L	388 263 621 2334	MSV-N/2 × 10^−18^/72/27.58 no hits no hits MSV-L/0.0/100/39.64
Hueffelii catchfly (*Silene heuffelii*)	Dicot/ *Caryophyllaceae*	Silene lispi-like virus 1_heu/ SilLlV1_heu	PRJEB39526/ [133]	12347/128.39X	BK075350	N P2 P3 L	388 263 621 2334	MSV-N/2 × 10^−18^/72/27.58 no hits no hits MSV-L/0.0/100/39.64
bladder campion (*Silene vulgaris*)	Dicot/ *Caryophyllaceae*	Silene lispi-like virus 2/ SilLlV2	PRJEB39526/ [133]	12174/60.44X	BK075351	N P2 P3 L	390 250 626 2334	MSV-N/2 × 10^−21^/73/28.32 no hits no hits MSV-L/0.0/99/38.93
Limpricht’s Sinalliaria (*Sinalliaria limprichtiana*)	Dicot/ *Brassicacea*	Sinalliaria lispi-like virus 1/ SinLlV1	PRJNA428441/ [134]	12804/45.07X	BK075352	N P2 P3 L	407 232 587 2375	MSV-N/7 × 10^−19^/6/25.09 no hits no hits MSV-L/0.0/100/37.44
wild mustard (*Sinapis arvensis*)	Dicot/ *Brassicacea*	Sinapis lispi-like virus 1/ SinaLlV1	PRJNA232677/ [135]	12112/61.46X	BK075353	N P2 P3 L	385 233 601 2342	MSV-N/1 × 10^−24^/86/26.39 no hits no hits MSV-L/0.0/99/39.88
Katherine sorghum (*Sorghum macrospermum*)	Monocot/ *Poaceae*	Sorghum lispi-like virus 1/ SorLlV1	PRJNA736757/ [136]	12155/434.64X	BK075354	N P2 P3 L	374 230 623 2335	MSV-N/8 × 10^−43^/83/29.94 no hits no hits MSV-L/0.0/100/41.46
narrow-saline sipweed (*Suaeda salsa*)	Dicot/ *Amaranthaceae*	Suaeda lispi-like virus 1/ SuaLlV1	PRJNA383092/ Zheng, M., FIO, China, unpublished	12704/13.26X	BK075355	N P2 P3 L	390 239 699 2365	MSV-N/2 × 10^−31^/69/31.34 no hits no hits MSV-L/0.0/98/36.93
sunflower (*Helianthus annuus*)	Dicot/ *Asteraceae*	sunflower lispi-like virus 1/ SunLlV1	PRJNA408292/ [137]	13579/19.07X	BK075356	N P2 P3 L	393 215 599 2341	MSV-N/1 × 10^−13^/54/25.57 no hits no hits MSV-L/0.0/100/34.38
Dalmatian chrysanthemum (*Tanacetum cinerariifolium*)	Dicot/ *Asteraceae*	Tanacetum lispi-like virus 1/ TanLlV1	PRJNA399494/ [138]	11778/12.27X	BK075357	N P2 P3 L	368 221 663 2338	MSV-N/4 × 10^−37^/75/30.58 no hits no hits MSV-L/0.0/100/45.14
bull clover (*Trioflium fucatum*)	Dicot/ *Fabaceae*	Trifolium lispi-like virus 1/ TriLlV1	PRJNA311722/ [139]	12046/97.71X	BK075358	N P2 P3 L	374 225 609 2391	MSV-N/5 × 10^−41^/75/30.36 no hits no hits MSV-L/0.0/98/45.49
broad bean (*Vicia faba*)	Dicot/ *Fabaceae*	Vicia lispi-like virus 1/ VicLlV1	PRJNA679419/ [140]	13583/122.89X	BK075359	N P2 P3 L	431 253 661 2342	MSV-N/2 × 10^−14^/56/26.72 no hits no hits MSV-L/0.0/100/36.32
Korean mistletoe (*Viscum coloratum*)	Dicot/ *Santalaceae*	Viscum lispi-like virus 1/ VisLlV1	PRJNA350768/ [141]	11348/28.59X	BK075360	N P2 P3 L	370 219 606 2343	MSV-N/1 × 10^−31^/86/28.83 no hits no hits MSV-L/0.0/100/39.26

**Table 2:** Consensus conserved lispi-like viruses gene junction sequences.

Virus *	3′end mRNA	Intergenic Spacer	5′end mRNA
MSuV	AUCUUUUUU	CAC	UCU
AgeLlV1	AAUAUUUUU	CAC	UUG
AgeLlV2	UUAUUUUUU	CAC	UCC
AllLlV1	AUCUUUUUU	CAC	UCC
AllLlV2	AACUUUUUU	CAC	UUU
AllLlV3	AUCUUUUUU	CAC	UGG
AmbLlV1	AAAUUUUUU	CAC	UUU
AraLlV1	AUUCUUUUU	CGC	UCU
ArcLlV1	AUCUUUUUU	CAU	UCU
ArnLlV1	AUCUUUUUU	CAC	UCU
AstLlV1	AAAUUUUUU	CAC	UCU
AtrLlV1	UUAUUUUUU	CAC	UCU
BraLlV1	AUAUUUUUU	CUC	UUU
BraLlV2	UAAUUUUUU	CAC	UUG
BraLlV3	AAUCUUUUU	CGC	UCC
CalLlV1	AUCUUUUUU	CAC	UCC
CalLlV2	AAUCUUUUU	CUC	UCU
CanLlV1	AUCUUUUUU	CAC	UCC
CapLlV1	AUUCUUUUU	CGC	UUC
CarLlV1	AAAUUUUUU	CAC	UCG
CenLlV1	AUAUUUUUU	CAC	UCC
CentLlV1	AUAUUUUUU	CAC	UCG
CicLlV1	AUUAUUUUU	CAC	UUU
CisLlV1	AUCUUUUUU	CGC	UCU
CisLlV2	UACUUUUUU	CGC	UUG
CopLlV1	AUCUUUUUU	CAC	UCC
CorLlV1	AAAUUUUUU	CGC	UUU
CorLlV2	AUCUUUUUU	CAC	UCU
CusLlV1	UAAUUUUUU	CAC	UCU
CypLlV1	CGUUUUUUU	CGC	UCU
CypLlV2	AUGUUUUUU	CAC	UCC
DacLlV1	AACUUUUUU	CAC	UCU
DacLlV2	AUCUUUUUU	CGC	UCC
DacLlV3	AUCUUUUUU	CAC	UCU
DioLlV1	UUGUUUUUU	CAC	UCC
EpiLlV1	UUCUUUUUU	CAC	UCU
EpiLlV2	UAAUUUUUU	CGC	UCC
EriLlV1	UUAUUUUUU	CAC	UCC
EruLlV1	AUAUUUUUU	CAC	UUA
EryLlV1	AUAUUUUUU	CGC	UUA
GuiLlV1	AUCUUUUUU	CGC	UCC
GymLlV1	AUCUUUUUU	CAC	UCU
HelLlV1	AUAUUUUUU	CAU	UCA
HerLlV1	AUCUUUUUU	CAC	UCU
IxiLlV1	AUUCUUUUU	CGC	UCU
LacLlV1	AUCUUUUUU	CAC	UCG
LimLlV1	AUAUUUUUU	CAC	UUC
MerLlV1	AUAUUUUUU	CAC	UCA
MonLlV1	AUCUUUUUU	CGC	UCU
MorLlV1	AUAUUUUUU	CAC	UCU
OphLlV1	AUUCUUUUU	CAC	UCU
OphLlV2	AUUCUUUUU	CAC	UCU
OryLlV1	AUUCUUUUU	CAC	UCC
ParLlV1	AUCUUUUUU	CAC	UCC
PhaLlV1	AUGUUUUUU	CAC	UCU
PlaLlV1	AAAUUUUUU	CAU	UCU
RapLlV1	AUUCUUUUU	CGC	UCC
RhoLlV1	AUCUUUUUU	CGC	UCU
RhoLlV2	AUCUUUUUU	CGC	UCU
RhoLlV3	UUGUUUUUU	CGC	UCU
RhoLlV4	UUAUUUUUU	CGC	UCU
RhoLlV5	AUAUUUUUU	CGC	UCU
RhoLlV6	AUAUUUUUU	CAC	UCU
RhoLlV7	AUCUUUUUU	CGC	UCU
RumLlV1	AUAUUUUUU	CGC	UCU
RumLlV2	AACUUUUUU	CAC	UCU
SapLlV1	AUAUUUUUU	CAC	UCU
ScaLlV1	AUGUUUUUU	CAC	UCU
SilLlV1	AUUCUUUUU	CGC	UCU
SilLlV2	AUUCUUUUU	CGC	UCU
SinLlV1	UUCUUUUUU	CGC	UCU
SinaLlV1	AUUCUUUUU	CAC	UCC
SorLlV1	AUCUUUUUU	CAC	UCC
SuaLlV1	AUCUUUUUU	CGC	UCC
SunLlV1	AUAAUUUUU	CUC	UCG
TanLlV1	AUCUUUUUU	CAC	UCC
TriLlV1	AUCUUUUUU	CAC	UCC
VicLlV1	UAAUUUUUU	CAC	UCG
VisLlV1	AUUCUUUUU	CAC	UCU

The consensus gene junction sequences of the viruses identified in this study are highlighted in light grey. * Names and abbreviations of newly identified viruses are listed in Table 1; MSuV stands for maize suscal virus.

### 3.14. Implications for Viral Ecology and Evolution

The comprehensive view of lispi-like virus diversity emerging from this data-mining effort reveals several key ecological and evolutionary patterns. First, extreme host specialization predominates despite substantial sampling, suggesting that molecular barriers to host range expansion are significant and that generalist viruses represent rare evolutionary achievements rather than common outcomes. Second, the viruses capable of generalist lifestyles show no consistent phylogenetic clustering, suggesting that host range expansion has evolved multiple times independently through convergent molecular adaptations.

Third, the strong correlation between agricultural importance/research intensity and virus detection highlights how anthropogenic factors shape our knowledge of viral diversity. Wild plant viruses remain vastly undersampled, representing an unknown risk for agricultural emergence and a substantial reservoir of unexplored biodiversity. Finally, the accelerating discovery rate indicates that plant virus diversity remains substantially underestimated even in well-studied systems. As sequencing costs continue declining and sampling extends into previously neglected plant groups and geographic regions, we anticipate continued discovery of novel viral lineages that will reshape our understanding of plant virome diversity, evolution, and ecology.

### 3.15. Strengths and Limitations of Sequence Discovery Through Data Mining

As previously demonstrated by Bejerman and colleagues [11,42], independent validation through re-analysis of SRA data can significantly enhance our understanding of RNA virus genomes with unique features. However, a key limitation of this approach is the lack of access to original biological material, which impedes replication and verification of the assembled viral genome sequences, an inherent weakness of data mining-based virus discovery. Additionally, potential issues such as contamination, low sequencing quality, sample spillover, and other technical artifacts present a risk of generating false-positive, chimeric assemblies, or incorrect host assignments. Consequently, caution must be exercised when interpreting results derived from publicly available SRA data. To strengthen and validate our findings, we strongly recommend generating new RNA-seq datasets from the predicted plant hosts. While our approach has inherent limitations, some aspects of our virus discovery strategy, such as iterative contig extension and comprehensive sequence validation, can aid to alleviate some of these challenges and provide further evidence for identification. Nevertheless, it is key to emphasize that all virus–host associations and detections should be considered preliminary until confirmed through further investigation.

### 3.16. Excluding Insect-Derived and Cross-Library Contamination

Sequence-based virus discovery from public plant transcriptomes must also exclude the possibility that the viruses recovered are not plant-associated at all, but derive instead from arthropods present on the sampled material or from cross-contamination between multiplexed libraries. Several independent lines of evidence argue against this interpretation for the masuviruses. First, the taxonomic composition of the source libraries is overwhelmingly plant. STAT k-mer taxonomic profiles were obtained for representative runs of 66 of the 67 independent BioProjects underlying Table 1; across these runs a median of 72.8% of taxonomically identified reads were assigned to Viridiplantae (range 1.5–99.2%), whereas a median of only 0.009% were assigned to Arthropoda (mean 0.15%, maximum 2.85%) and 0.004% to Insecta (Supplementary Figure S8; Supplementary Table S2). Arthropod-assigned reads fell below 0.1% of identified reads in 56 of 66 runs and below 0.01% in 33 runs, and were entirely absent in five; the median ratio of plant- to arthropod-assigned reads was approximately 4.8 × 10^3^. In the minority of libraries in which plant reads are not the dominant assigned fraction, the remainder is bacterial, fungal, or chordate rather than arthropod, so arthropod content is negligible throughout the dataset irrespective of overall library composition. Recovering coding-complete genomes of 11.2–13.6 kb at a median depth of 64-fold coverage (range 12–909-fold; Table 1) from a library component represented at ~0.009% of identified reads is not quantitatively plausible: under an arthropod-carryover model the virus would have to be present in the putative arthropod source at a titre several orders of magnitude above that of the carried-over arthropod transcripts themselves, in every one of 67 independent projects.

Second, and more decisively, contamination cannot generate the evolutionary structure that is observed. The 87 sequences form a single monophyletic lineage that is sister to, and not nested within, the invertebrate-associated *Lispiviridae* (Figure 1B). Arthropod carryover samples the donor virome essentially at random and would therefore be expected to deposit sequences that fall among the known insect-associated lispiviruses; instead, every sequence recovered from a plant library falls within one exclusive clade whose entire membership is plant-derived. Third, this clade is defined by a shared derived genome architecture that differs from that of the *Lispiviridae*: all 87 sequences share the four-cistron organisation 3′-N(P1)-P2-P3-L(P4)-5′ and lack the glycoprotein cistron typical of lispivirids, and all share a family-wide gene-junction grammar comprising a U-rich terminator, an invariant CAC or CGC trinucleotide spacer and a conserved UCU or UCC initiator (Table 2). A contaminating insect virus would carry the architecture of its own lineage. Fourth, divergence within the clade is structured by plant taxonomy rather than by technical provenance: L-protein identities are appreciably higher between viruses sharing a host botanical family than between viruses from different host families (mean 50.5% versus 38.8%; Supplementary Figure S1B), and several proposed genera show complete host-family fidelity, including Betamasuvirus (*Brassicaceae*), Nu-, Kappa- and Lambdamasuvirus (*Orchidaceae*) and Epsilonmasuvirus, whose seven isolates all derive from a single Balanophoraceae species. Contamination, whether arthropod-derived or between libraries, tracks sequencing batch, facility and submitter, and has no mechanism by which to reproduce botanical-family structure. Fifth, detection is independent and repeated: the 87 sequences derive from 67 independent BioProjects, and the retrospective Logan screen recovered the lineage in 1536 libraries representing 134 plant species from 182 geographic locations and nine years of deposition. Index hopping and cross-contamination are within-run or within-facility phenomena and cannot be reproduced coherently across unrelated submitters, continents, years, and library chemistries; that Brassica lispi-like virus 3 remains confined to *Brassicaceae* across 172 libraries, and Brassica lispi-like virus 2 across 509 libraries derived from 72 independent studies, is incompatible with a contaminant, which would not respect a botanical boundary. Sixth, several hosts are ecologically implausible sources of a shared insect contaminant, including the holoparasitic *Rhopalocnemis phalloides* and mycoheterotrophic and orchid hosts sampled on different continents, and detections include root, seed and flower tissues rather than being confined to the aerial tissues on which arthropods would be expected. Finally, the founding member of the lineage, MSuV, was characterised from plant material, forms rod-shaped virions in maize tissue and is seed-transmitted [28], so the clade is anchored by a virus experimentally established as plant-infecting.

We nonetheless emphasise the limit of what these arguments establish. Together they support a genuine and specific association between these viruses and the plants in whose transcriptomes they occur, and they exclude arthropod carryover and inter-library contamination as a general explanation for the dataset; they do not, by themselves, demonstrate replication within plant cells for any individual virus. Definitive evidence of plant infection requires data that cannot be obtained from archived libraries, most directly the detection of negative-sense replicative intermediates by strand-specific RT-PCR, the recovery of virus-derived small interfering RNAs of the size classes generated by plant antiviral RNA silencing, or in situ localisation in plant tissue. These remain the appropriate next steps, and, consistent with the caveats set out above, all virus–host associations reported here should be regarded as provisional until so confirmed.

## 4. Conclusions

Our study reinforces and highlights the key importance of analyzing SRA public data as a valuable tool, not only for expediting the discovery of novel viruses but also for gaining insights into their evolutionary history and enhancing virus classification. Through this approach, we conducted a search for hidden lispi-like virus sequences, which significantly expanded the number of these viruses. Our analysis allowed us to shed light into its taxonomical classification, allocating a new plant-associated group of viruses to a new family within the *Mononegavirales* order; thus, being just the second family within this order that includes plant-associated members, which highlights their diversity and complex evolutionary dynamics of mononegaviruses. Some biological properties, such as transmission and viral particle morphology, were characterized for MSuV [28]; however, for all the other members, only the sequence was characterized. With the increasing reliance on sequence-based taxonomy, current classifications primarily reflect evolutionary relationships inferred from phylogenetic analyses of the L gene. In this context, the MSuV biological and sequence properties, as well as the sequence properties of all newly identified viruses in our study, together with our phylogenetic analyses, support the assignment of these viruses within a new family, here proposed Masuviridae, within the order *Mononegavirales*.

## Data Availability

Nucleotide sequence data reported are available in the Third Party Annotation Section of the DDBJ/ENA/GenBank databases under the accession numbers TPA: BK075274-BK075360 and can be found as Supplementary Material S1 of this submission.

## Supplementary Materials

The following supporting information can be downloaded at: https://www.mdpi.com/article/10.3390/v18101113/s1. Supplementary Materials S1. Virus sequences described in this study. Supplementary Figure S1. *Sequence-level characterisation of the L (P4) polymerase across 87 lispi-like viruses.* (A) Distribution of pairwise amino-acid identities, partitioned into within-host-family (blue) and between-host-family (grey) pairs (median 38.5%). (B) Pairwise identity matrix ordered by host botanical family (colour bar), showing coherent within-family blocks. (C) Per-site conservation (1 − H/H_max; 21-column sliding window) along the P4 alignment; the structurally resolved RdRp core (mean 0.69) is significantly more conserved than the structurally unresolved capping/methyltransferase/C-terminal region (mean 0.49; one-sided Mann–Whitney U, *p* ≈ 2 × 10^−65^). Vertical marks indicate catalytic motifs A, B and C. (D) Sequence logos (information content, bits; n = 87) for motifs A, B and C. (E) Per-position percentage identity across the palmprint; catalytic residues (motif-A aspartate; motif-C G-D-N) in red. (F) Summary of catalytic-residue conservation: motif-A aspartate 100% (DFSKW invariant), motif-C G-D-N 100% (GDN in 87/87; no GDD or SDD variant), motif-B block GWEGQRQKGW 95.4% (83/87). The uniform GDN signature places P4 in the *Mononegavirales*/*Rhabdoviridae* RdRp catalytic class. Supplementary Figure S2. *Impact of Normalization on Virus Ranking* Comparison of virus prevalence rankings using raw library counts (A) versus normalized Virus Prevalence Score (VPS) (B). VPS is calculated as (Unique Hosts/√Libraries) × 100. Color gradients indicate detection frequency, with normalization revealing viruses that infect diverse hosts despite fewer total detections. Supplementary Figure S3. *Host Diversity versus Library Abundance* Scatter plot examining the relationship between total SRA libraries (*x*-axis, log scale) and number of unique host organisms (*y*-axis) for detected viruses. Bubble size represents normalized prevalence score (VPS), while color intensity indicates detection robustness (proportion of libraries where virus was detected). Supplementary Figure S4. *Library Redundancy Analysis* Comparison of total SRA libraries versus unique independent BioProjects for the top 30 viruses. Blue bars represent total library counts, while orange bars show unique BioProjects. Supplementary Figure S5. *Host Range Distribution: Monocots versus Dicots* Violin plots comparing the number of unique host organisms for dicot-dominated versus monocot-specific viruses. Supplementary Figure S6. *Tissue Tropism Patterns in Top 25 Viruses* Stacked bar chart showing proportional distribution of tissue types from which each virus was detected. Viruses are ordered by total detection frequency (left to right). Supplementary Figure S7. *Global Distribution of Viral Detections* World map showing 182 unique sampling locations across 252 countries. Bubble size indicates number of samples per location, while color represents virus richness (number of distinct viruses detected). Supplementary Figure S8. Taxonomic composition of the source sequencing libraries. Percentage of taxonomically identified reads assigned to Viridiplantae (green) and to Arthropoda (orange) in representative runs from 66 of the 67 independent BioProjects underlying Table. Supplementary Table S1. Summary of dataset counts at each stage of sequence identification, filtering and classification. All counts reported elsewhere in the manuscript, in the figures and in Table 1 and Table 2 refer back to this table. Supplementary Table S2. SRA Taxonomy Analysis Tool (STAT) taxonomic composition of the source sequencing libraries. For each of the 66 independent BioProjects contributing sequences to Table 1 for which a precomputed STAT profile was available, a representative run is shown. The run shown represents its BioProject and is not necessarily the specific run from which the viral contig was assembled. The one remaining project, PRJNA763316, has no precomputed STAT profile for any of its runs. Percentages are expressed relative to the total number of taxonomically identified reads in that run. Plant: arthropod is the ratio of Viridiplantae-assigned to Arthropoda-assigned reads (computed with a pseudocount of 1 to accommodate runs with no arthropod-assigned reads).

## References

[1] Koonin E.V., Dolja V.V., Krupovic M., Kuhn J.H. (2021). Viruses Defined by the Position of the Virosphere within the Replicator Space. Microbiol. Mol. Biol. Rev..

[2] Koonin E., Kuhn J., Dolja V., Krupovic M. (2024). Megataxonomy and global ecology of the virosphere. ISME J..

[3] Geoghegan J.L., Holmes E.C. (2017). Predicting virus emergence amid evolutionary noise. Open Biol..

[4] Dominguez-Huerta G., Wainaina J.M., Zayed A.A., Culley A.I., Kuhn J.H., Sullivan M.B. (2023). The RNA virosphere: How big and diverse is it?. Environ. Microbiol..

[5] Hou X., He Y., Fang P., Mei S., Xu Z., Wu W.-C., Tian J.-H., Zhang S., Zeng Z.-Y., Gou Q.-Y. (2024). Using artificial intelligence to document the hidden RNA virosphere. Cell.

[6] Lu C., Wu Y., Zhang Z., Mao L., Ge X., Wu A., Sun F., Jiang Y., Peng Y. (2025). Estimated size of the total genome and protein space of viruses. mSphere.

[7] Dolja V.V., Krupovic M., Koonin E.V. (2020). Deep roots and splendid boughs of the global plant virome. Annu. Rev. Phytopathol..

[8] Edgar R.C., Taylor J., Lin V., Altman T., Barbera P., Meleshko D., Lohr D., Novakovsky G., Buchfink B., Al-Shayeb B. (2022). Petabase-scale sequence alignment catalyses viral discovery. Nature.

[9] Koonin E.V., Dolja V.V., Krupovic M. (2022). The logic of virus evolution. Cell Host Microbe.

[10] Mifsud J.C., Gallagher R.V., Holmes E.C., Geoghegan J.L. (2022). Transcriptome Mining Expands Knowledge of RNA Viruses across the Plant Kingdom. J. Virol..

[11] Bejerman N., Dietzgen R., Debat H. (2023). Novel Tri-Segmented Rhabdoviruses: A Data Mining Expedition Unveils the Cryptic Diversity of ytorhabdoviruses. Viruses.

[12] Charon J., Olendraite I., Forgia M., Chong L.C., Hillary L.S., Roux S., Kupczok A., Debat H., Sakaguchi S., Tahzima R. (2024). Consensus statement from the first RdRp Summit: Advancing RNA virus discovery at scale across communities. Front. Virol..

[13] Sidharthan V.K., Baranwal V.K. (2024). Public domain databases: A gold mine for identification and genome reconstruction of plant viruses and viroids. Genomics Data Analysis for Crop Improvement.

[14] Liu Q., Gong Z., Koonin E.V., Dolja V.V., Han G.Z. (2026). The Origins and Evolution of the RNA Virome in Land Flora. Mol. Plant.

[15] Lauber C., Seitz S. (2022). Opportunities and challenges of data-driven virus discovery. Biomolecules.

[16] Simmonds P., Adams M.J., Benkő M., Breitbart M., Brister J.R., Carstens E.B., Davison A.J., Delwart E., Gorbalenya A.E., Harrach B. (2017). Virus taxonomy in the age of metagenomics. Nat. Rev. Microbiol..

[17] Simmonds P., Adriaenssens E.M., Zerbini F.M., Abrescia N.G.A., Aiewsakun P., Alfenas-Zerbini P., Bao Y., Barylski J., Drosten C., Duffy S. (2023). Four principles to establish a universal virus taxonomy. PLoS Biol..

[18] Gao Z., Wu J., Lucaci A.G., Ouyang J., Wang L., Ryon K.A., Elhaik E., Probst A.J., Rodó X., Velavan T.P. (2026). Diversity and distinctive characteristics of the global RNA virome in urban and peri-urban environments. Nat. Commun..

[19] Debat H., Garcia M.L., Bejerman N. (2023). Expanding the Repertoire of the Plant-Infecting Ophioviruses through Metatranscriptomics Data. Viruses.

[20] Marra M., Rotunno S., Frascati F., Pierro R., Hammond J., Vaira A.M., Miozzi L. (2026). The analysis of the virome associated with Freesia refracta plants with necrotic disorder sheds new light on the phylogenetic relationships in the Konkoviridae and Yueviridae families. Virol. J..

[21] Nomburg J., Doherty E.E., Price N., Bellieny-Rabelo D., Zhu Y.K., Doudna J.A. (2024). Birth of protein folds and functions in the virome. Nature.

[22] Mifsud J.C., Suchard M.A., Holmes E.C., Lemey P. (2025). Recent advances in the inference of deep viral evolutionary history. J. Virol..

[23] Mutz P., Camargo A.P., Sahakyan H., Neri U., Butkovic A., Wolf Y.I., Krupovic M., Dolja V.V., Koonin E.V. (2025). The protein structurome of Orthornavirae and its dark matter. mBio.

[24] Odai R., Leemann M., Al-Murad T., Abdullah M., Shyrokova L., Tenson T., Hauryliuk V., Durairaj J., Pereira J., Atkinson G.C. (2025). The Viral AlphaFold Database of monomers and homodimers reveals conserved protein folds in viruses of bacteria, archaea, and eukaryotes. Sci. Adv..

[25] Amarasinghe G.K., Ayllon M.A., Bao Y., Basler C.F., Bavari S., Blasdell K.R., Briese T., Brown P.A., Bukreyev A., Balkema-Buschmann A. (2019). Taxonomy of the order Mononegavirales: Update 2019. Arch. Virol..

[26] Walker P.J., Bejerman N., Blasdell K.R., Debat H., Dietzgen R.G., Fooks A.R., Freitas-Astúa J., Garver K., Kondo H., Ramos-González P.L. (2026). ICTV Virus Taxonomy Profile: Rhabdoviridae 2026. J. Gen. Virol..

[27] Li J.M., Wang F., Ye G., Paraskevopoulou S. (2023). ICTV Virus Taxonomy Profile: Lispiviridae 2023. J. Gen. Virol..

[28] Alvarez-Quinto R.A., Quito-Avila D.F., Cornejo J.F., Grinstead S., Mollov D., Lockhart B.E. (2021). A novel rod-shaped virus isolated from maize in Ecuador. Phytopathology.

[29] Li Q., Yang L., Zhu T., Yv X., Zhang B., Li H., Hao J., Zhang L., Ji P., Dong J. (2024). Detection and discovery of plant viruses in Disporopsis through high-throughput sequencing. Front. Microbiol..

[30] Bolger A.M., Lohse M., Usadel B. (2014). Trimmomatic: A flexible trimmer for Illumina sequence data. Bioinformatics.

[31] Teufel F., Armenteros J.J.A., Johansen A.R., Gíslason M.H., Pihl S.I., Tsirigos K.D., Winther O., Brunak S., von Heijne G., Nielsen H. (2022). SignalP 6.0 Predicts All Five Types of Signal Peptides Using Protein Language Models. Nat. Biotechnol..

[32] Käll L., Krogh A., Sonnhammer E.L. (2007). Advantages of combined transmembrane topology and signal peptide prediction, the Phobius web server. Nucleic Acids Res..

[33] Van Kempen M., Kim S.S., Tumescheit C., Mirdita M., Lee J., Gilchrist C.L.M., Söding J., Steinegger M. (2024). Fast and accurate protein structure search with Foldseek. Nat. Biotechnol..

[34] Katoh K., Standley D.M. (2013). MAFFT multiple sequence alignment software version 7: Improvements in performance and usability. Mol. Biol. Evol..

[35] Muhire B.M., Varsani A., Martin D.P. (2014). SDT: A virus classification tool based on pairwise sequence alignment and identity calculation. PLoS ONE.

[36] Tamura K., Stecher G., Kumar S. (2021). MEGA11: Molecular Evolutionary Genetics Analysis Version 11. Mol. Biol. Evol..

[37] Jumper J., Evans R., Pritzel A., Green T., Figurnov M., Ronneberger O., Tunyasuvunakool K., Bates R., Žídek A., Potapenko A. (2021). Highly accurate protein structure prediction with AlphaFold. Nature.

[38] Evans R., O’Neill M., Pritzel A., Antropova N., Senior A., Green T., Žídek A., Bates R., Blackwell S., Yim J. (2022). Protein complex prediction with AlphaFold-Multimer. bioRxiv.

[39] Liang B., Li Z., Jenni S., Rahmeh A.A., Morin B.M., Grant T., Grigorieff N., Harrison S.C., Whelan S.P.J. (2015). Structure of the L protein of vesicular stomatitis virus from electron cryomicroscopy. Cell.

[40] Nei M., Gojobori T. (1986). Simple methods for estimating the numbers of synonymous and nonsynonymous nucleotide substitutions. Mol. Biol. Evol..

[41] Chikhi R., Lemane T., Loll-Krippleber R., Montoliu-Nerin M., Raffestin B., Camargo A.P., Miller C.J., Fiamenghi M.B., Agustinho D.P., Majidian S. (2025). Logan: Planetary-scale genome assembly surveys life’s diversity. bioRxiv.

[42] Bejerman N., Dietzgen R.G., Debat H. (2022). Unlocking the hidden genetic diversity of varicosaviruses, the neglected plant rhabdoviruses. Pathogens.

[43] Bejerman N., Dietzgen R.G., Debat H. (2026). Expanding the known nucleorhabdovirus world: The final chapter in a trilogy exploring the hidden diversity of plant-associated rhabdoviruses. Virology.

[44] Bejerman N., Dietzgen R., Debat H. (2021). Illuminating the Plant Rhabdovirus Landscape through Metatranscriptomics Data. Viruses.

[45] Latorre V., Mattenberger F., Geller R. (2018). Chaperoning the Mononegavirales: Current Knowledge and Future Directions. Viruses.

[46] Liang B. (2020). Structures of the Mononegavirales Polymerases. J. Virol..

[47] Luo M., Terrell J.R., McManus S.A. (2020). Nucleocapsid Structure of Negative Strand RNA Virus. Viruses.

[48] Green T.J., Cox R., Tsao J., Rowse M., Qiu S., Luo M. (2014). Common mechanism for RNA encapsidation by negative-strand RNA viruses. J. Virol..

[49] Luo M., Lyles K., Faniyi O., Kim R. (2026). Assembly of the linear viral nucleocapsid. Microorganisms.

[50] Ker D.S., Jenkins H.T., Greive S.J., Antson A.A. (2021). CryoEM structure of the Nipah virus nucleocapsid assembly. PLoS Pathog..

[51] Illergård K., Ardell D.H., Elofsson A. (2009). Structure is three to ten times more conserved than sequence, a study of structural response in protein cores. Proteins.

[52] Galperin M.Y., Moroz O.V., Wilson K.S., Murzin A.G. (2006). House cleaning, a part of good housekeeping. Mol. Microbiol..

[53] Rost B. (1999). Twilight zone of protein sequence alignments. Protein Eng..

[54] Eddy S.R. (2011). Accelerated profile HMM searches. PLoS Comput. Biol..

[55] Mistry J., Chuguransky S., Williams L., Qureshi M., Salazar G.A., Sonnhammer E.L.L., Tosatto S.C., Paladin L., Raj S., Richardson L.J. (2021). Pfam: The protein families database in 2021. Nucleic Acids Res..

[56] Uversky V.N., Gillespie J.R., Fink A.L. (2000). Why are “natively unfolded” proteins unstructured under physiologic conditions?. Proteins Struct. Funct. Bioinform..

[57] Valli A.A., López R.G., Ribaya M., Martínez F.J., Gómez D.G., García B., Gonzalo I., de Prádena A.G., Pasin F., Montanuy I. (2022). Maf/ham1-like pyrophosphatases of non-canonical nucleotides are host-specific partners of viral RNA-dependent RNA polymerases. PLoS Pathog..

[58] Schroader J.H., Handley M.T., Reddy K. (2023). Inosine triphosphate pyrophosphatase: A guardian of the cellular nucleotide pool and potential mediator of RNA function. WIREs RNA.

[59] Tomlinson K.R., Pablo-Rodriguez J.L., Bunawan H., Nanyiti S., Green P., Miller J., Alicai T., Seal S.E., Bailey A.M., Foster G.D. (2019). Cassava brown streak virus Ham1 protein hydrolyses mutagenic nucleotides and is a necrosis determinant. Mol. Plant Pathol..

[60] Fernández I., Bontems F., Brun D., Coquin Y., Goverde C., Coreilla B.E., Gessain A., Buseyne F., Rey F.A., Backovic M. (2024). Structure of the foamy virus envelope glycoprotein in its prefusion and postfusion states. Sci. Adv..

[61] Chao L.H., Jang J., Johnson A., Nguyen A., Gray N.S., Yang P.L., Harrison S.C. (2015). Viral membrane fusion. Virology.

[62] White J.M., Delos S.E., Brecher M., Schornberg K. (2008). Structures and mechanisms of viral membrane fusion proteins: Multiple variations on a common theme. Crit. Rev. Biochem. Mol. Biol..

[63] Kosakovsky Pond S.L., Frost S.D.W. (2005). Not so different after all: A comparison of methods for detecting amino acid sites under selection. Mol. Biol. Evol..

[64] Poch O., Sauvaget I., Delarue M., Tordo N. (1989). Identification of four conserved motifs among the RNA-dependent RNA polymerase encoding elements. EMBO J..

[65] Kuhn J.H., Wolf Y.I., Krupovic M., Zhang Y.-Z., Maes P., Dolja V.V., Koonin E.V. (2019). Classify viruses—the gain is worth the pain. Nature.

[66] Babaian A., Edgar R. (2022). Ribovirus classification by a polymerase barcode sequence. PeerJ.

[67] Venkataraman S., Prasad B.V., Selvarajan R. (2018). RNA dependent RNA polymerases: Insights from structure, function and evolution. Viruses.

[68] Sánchez A.B., de la Torre J.C. (2005). Genetic and biochemical evidence for an oligomeric structure of the functional L polymerase of the prototypic arenavirus lymphocytic choriomeningitis virus. J. Virol..

[69] Tomar S., Hardy R., Smith J., Kuhn R. (2006). Catalytic core of alphavirus nsP4 possesses terminal adenylyltransferase activity. J. Virol..

[70] Chattopadhyay A., Raha T., Shaila M.S. (2004). Effect of single amino acid mutations in the conserved GDNQ motif of L protein of Rinderpest virus on RNA synthesis in vitro and in vivo. Virus Res..

[71] Paesen G.C., Collet A., Sallamand C., Debart F., Vasseur J.J., Canard B., Decroly E., Grimes J.M. (2015). X-ray structure and activities of an essential Mononegavirales L-protein domain. Nat. Commun..

[72] Malur A.G., Gupta N.K., De Bishnu P., Banerjee A.K. (2002). Analysis of the mutations in the active site of the RNA-dependent RNA polymerase of human parainfluenza virus type 3 (HPIV3). Gene Expr..

[73] Neubauer J., Ogino M., Green T.J., Ogino T. (2015). Signature motifs of GDP polyribonucleotidyltransferase, a non-segmented negative strand RNA viral mRNA capping enzyme, domain in the L protein are required for covalent enzyme-pRNA intermediate formation. Nucleic Acids Res..

[74] Ogino T., Green T.J. (2019). RNA synthesis and capping by non-segmented negative strand RNA viral polymerases. Front. Microbiol..

[75] Hasiów-Jaroszewska B., Boezen D., Zwart M.P. (2021). Metagenomic Studies of Viruses in Weeds and Wild Plants: A Powerful Approach to Characterize Variable Virus Communities. Viruses.

[76] LaTourrette K., Garcia-Ruiz H. (2022). Determinants of Virus Variation, Evolution, and Host Adaptation. Pathogens.

[77] McLeish M.J., Fraile A., Garcia-Arenal F. (2019). Evolution of plant-virus interactions: Host range and virus emergence. Curr. Opin. Virol..

[78] Kamran A., Srivastava A.K., Farooq T., Hussain M.D., Khan M., Li X., Xie X. (2025). Host shifting dynamics in plant viruses and biotechnological frontiers for global food security. Plant Physiol. Biochem..

[79] White J.W., Boote K.J., Kimball B.A., Porter C., Salmeron M., Shelia V., Thorp K.R., Hoogenboom G. (2025). From field to analysis: Strengthening reproducibility and confirmation in research for sustainable agriculture. npj Sustain. Agric..

[80] Núñez-Muñoz L.A., Calderón-Pérez B., Ruiz-Medrano R., Xoconostle-Cázares B., de la Torre-Almaraz R. (2025). Viral Tropism in Plants, Reproductive Tissues, and Seeds. Arch. Microbiol..

[81] Groves C., German T., Dasgupta R., Mueller D., Smith D.L. (2016). Seed Transmission of Soybean Vein Necrosis Virus: The First Tospovirus Implicated in Seed Transmission. PLoS ONE.

[82] Lee D.-S., Kim J., Jun M., Shin S., Lee S.-J., Lim S. (2022). Complete genome sequence of a putative novel cytorhabdovirus isolated from *Rudbeckia* sp. Arch. Virol..

[83] Minutolo M., Nicoloso V., Cinque M., Chiumenti M., Simeone G.D.R., Di Serio F., Alioto D., Navarro B. (2024). A polyvalent tool for detecting coguviruses in multiple hosts allowed the identification of a novel seed-transmitted coguvirus infecting *Brassicaceae*. Phytopathology.

[84] Daru B.H. (2025). Tracking hidden dimensions of plant biogeography from herbaria. New Phytol..

[85] Skaldina O., Blande J.D. (2025). Global Biases in Ecology and Conservation Research: Insight from Pollinator Studies. Ecol. Lett..

[86] Jayasena A.S., Fisher M.F., Panero J.L., Secco D., Bernath-Levin K., Berkowitz O., Taylor N.L., Schilling E.E., Whelan J., Mylne J.S. (2017). Stepwise Evolution of a Buried Inhibitor Peptide over 45 My. Mol. Biol. Evol..

[87] Fajkus P., Peška V., Závodník M., Fojtová M., Fulnečková J., Dobias Š., Kilar A., Dvořáčková M., Zachová D., Nečasová I. (2019). Telomerase RNAs in land plants. Nucleic Acids Res..

[88] Feng B., Wang X., Chen S., Zhang Y., Su X., Song S. (2021). Transcriptome analysis and genetic diversity of *Allium victorialis* germplasms from the Changbai Mountains. Mitochondrial DNA B Resour..

[89] Virág E., Hegedűs G., Barta E., Nagy E., Mátyás K., Kolics B., Taller J. (2016). Illumina Sequencing of Common (Short) Ragweed (*Ambrosia artemisiifolia* L.) Reproductive Organs and Leaves. Front. Plant Sci..

[90] Wos G., Bohutínská M., Nosková J., Mandáková T., Kolář F. (2021). Parallelism in gene expression between foothill and alpine ecotypes in *Arabidopsis arenosa*. Plant J..

[91] Wang Y., Li X., Zhou W., Li T., Tian C. (2016). De novo assembly and transcriptome characterization of spruce dwarf mistletoe *Arceuthobium sichuanense* uncovers gene expression profiling associated with plant development. BMC Genom..

[92] Elliott A.G., Delay C., Liu H., Phua Z., Johan Rosengren K., Benfield A.H., Panero J.L., Colgrave M.L., Jayasena A.S., Dunse K.M. (2014). Evolutionary origins of a bioactive peptide buried within Preproalbumin. Plant Cell.

[93] Zhao Y., Zhang R., Jiang K.W., Qi J., Hu Y., Guo J., Zhu R., Zhang T., Egan A.N., Yi T.S. (2021). Nuclear phylotranscriptomics and phylogenomics support numerous polyploidization events and hypotheses for the evolution of rhizobial N-fixing symbiosis in Fabaceae. Mol. Plant.

[94] Zhang P., Zheng F., Chen L., Lu X., Tian W. (2020). CIP elicitors on the defense response of A. macrocephala and its related gene expression analysis. J. Plant Physiol..

[95] Tang X., Hao Y.J., Lu J.X., Lu G., Zhang T. (2019). Transcriptomic analysis reveals the mechanism of thermosensitive genic male sterility (TGMS) of *Brassica napus* under the high temperature inducement. BMC Genom..

[96] An H., Qi X., Gaynor M.L., Hao Y., Gebken S.C., Mabry M.E., McAlvay A.C., Teakle G.R., Conant G.C., Barker M.S. (2019). Transcriptome and organellar sequencing highlights the complex origin and diversification of allotetraploid *Brassica napus*. Nat. Commun..

[97] Peakall R., Wong D.C.J., Phillips R.D., Ruibal M., Eyles R., Rodriguez-Delgado C., Linde C.C. (2021). A multitiered sequence capture strategy spanning broad evolutionary scales: Application for phylogenetic and phylogeographic studies of orchids. Mol. Ecol. Resour..

[98] Xu H., Bohman B., Wong D.C.J., Rodriguez-Delgado C., Scaffidi A., Flematti G.R., Phillips R.D., Pichersky E., Peakall R. (2017). Complex Sexual Deception in an Orchid Is Achieved by Co-opting Two Independent Biosynthetic Pathways for Pollinator Attraction. Curr. Biol..

[99] Kryvokhyzha D., Milesi P., Duan T., Orsucci M., Wright S.I., Glémin S., Lascoux M. (2019). Towards the new normal: Transcriptomic convergence and genomic legacy of the two subgenomes of an allopolyploid weed (*Capsella bursa-pastoris*). PLoS Genet..

[100] Wang J., Haapalainen M., Nissinen A.I., Pirhonen M. (2021). Dual transcriptional profiling of carrot and ‘Candidatus Liberibacter solanacearum’ at different stages of infection suggests complex host-pathogen interaction. Mol. Plant-Microbe Interact..

[101] Deng C., Wang J., Lu C., Li Y., Kong D., Hong Y., Huang H., Dai S. (2020). CcMYB6-1 and CcbHLH1, two novel transcription factors synergistically involved in regulating anthocyanin biosynthesis in cornflower. Plant Physiol. Biochem..

[102] Testone G., Mele G., Di Giacomo E., Gonnella M., Renna M., Tenore G.C., Nicolodi C., Frugis G., Iannelli M.A., Arnesi G. (2016). Insights into the sesquiterpenoid pathway by metabolic profiling and de novo transcriptome assembly of stem-chicory (Cichorium intybus cultigroup “Catalogna”). Front. Plant Sci..

[103] Liu Y., Wang B., Shu S., Li Z., Song C., Liu D., Niu Y., Liu J., Zhang J., Liu H. (2021). Analysis of the Coptis chinensis genome reveals the diversification of protoberberine-type alkaloids. Nat. Commun..

[104] Wen J., Yu Y., Xie D.F., Peng C., Liu Q., Zhou S.D., He X.J. (2020). A transcriptome-based study on the phylogeny and evolution of the taxonomically controversial subfamily Apioideae (Apiaceae). Ann. Bot..

[105] Zhao J., Chen A.X., Gartrell R.D., Silverman A.M., Aparicio L., Chu T., Bordbar D., Shan D., Samanamud J., Mahajan A. (2019). Immune and genomic correlates of response to anti-PD-1 immunotherapy in glioblastoma. Nat. Med..

[106] Ruhlman T.A., Chang W.J., Chen J.J., Huang Y.T., Chan M.T., Zhang J., Liao D.C., Blazier J.C., Jin X., Shih M.C. (2015). NDH expression marks major transitions in plant evolution and reveals coordinate intracellular gene loss. BMC Plant Biol..

[107] Guo Y.-Y., Zhang Y.-Q., Zhang G.-Q., Huang L.-Q., Liu Z.-J. (2018). Comparative transcriptomics provides insight into the molecular basis of species diversification of section Trigonopedia (Cypripedium) on the Qinghai-Tibetan Plateau. Sci. Rep..

[108] Balao F., Trucchi E., Wolfe T.M., Hao B.-H., Lorenzo M.T., Baar J., Sedman L., Kosiol C., Amman F., Chase M.W. (2017). Adaptive sequence evolution is driven by biotic stress in a pair of orchid species (*Dactylorhiza*) with distinct ecological optima. Mol. Ecol..

[109] Bemm F., Becker D., Larisch C., Kreuzer I., Escalante-Perez M., Schulze W.X., Ankenbrand M., Van de Weyer A.L., Krol E., Al-Rasheid K.A. (2016). Venus flytrap carnivorous lifestyle builds on herbivore defense strategies. Genome Res..

[110] Lallemand F., Figura T., Damesin C., Fresneau C., Griveau C., Fontaine N., Zeller B., Selosse M.A. (2019). Mixotrophic orchids do not use photosynthates for perennial underground organs. New Phytol..

[111] Chen R.B., Liu J.H., Xiao Y., Zhang F., Chen J.F., Ji Q., Tan H.X., Huang X., Feng H., Huang B.K. (2015). Deep sequencing reveals the effect of MeJA on scutellarin biosynthesis in *Erigeron breviscapus*. PLoS ONE.

[112] Hao Y., Mabry M.E., Edger P.P., Freeling M., Zheng C., Jin L., VanBuren R., Colle M., An H., Abrahams R.S. (2021). The contributions from the progenitor genomes of the mesopolyploid Brassiceae are evolutionarily distinct but functionally compatible. Genome Res..

[113] Chen D., Liu Y., Pan Q., Li F., Zhang Q., Ge X., Li Z. (2018). De novo transcriptome assembly, gene expressions and metabolites for flower color variation of two garden species in *Brassicaceae*. Sci. Hortic..

[114] Osuna-Mascaró C., Rubio de Casas R., Landis J.B., Perfectti F. (2021). Genomic resources for *Erysimum* spp. (*Brassicaceae*): Transcriptome and chloroplast genomes. Front. Ecol. Evol..

[115] Gebeyehu A., Hammenhag C., Tesfaye K., Vetukuri R.R., Ortiz R., Geleta M. (2022). RNA-Seq provides novel genomic resources for noug (*Guizotia abyssinica*) and reveals microsatellite frequency and distribution in its transcriptome. Front. Plant Sci..

[116] Piñeiro Fernández L., Byers K.J.R.P., Cai J., Sedeek K.E.M., Kellenberger R.T., Russo A., Qi W., Aquino Fournier C., Schlüter P.M. (2019). A Phylogenomic Analysis of the Floral Transcriptomes of Sexually Deceptive and Rewarding European Orchids, Ophrys and Gymnadenia. Front. Plant Sci..

[117] Szukala A., Lovegrove-Walsh J., Luqman H., Fior S., Wolfe T.M., Frajman B., Schönswetter P., Paun O. (2022). Polygenic routes lead to parallel altitudinal adaptation in *Heliosperma pusillum* (Caryophyllaceae). Mol. Ecol..

[118] Liu S., Jiang X., Liu Z., Cheng Y., Sun T., Yu X. (2019). Mechanism of the Breaking of Seed Dormancy by Flower Thinning in Heracleum moellendorffii Hance. J. Plant Growth Regul..

[119] Unruh S.A., McKain M.R., Lee Y.-I., Yukawa T., McCormick M.K., Shefferson R.P., Smithson A., Leebens-Mack J.H., Pires J.C. (2018). Phylotranscriptomic analysis and genome evolution of the Cypripedioideae (*Orchidaceae*). Am. J. Bot..

[120] Oliya B.K., Kim M.Y., Lee S.H. (2018). Development of genic-SSR markers and genetic diversity of Indian lettuce (*Lactuca indica* L.) in South Korea. Genes Genom..

[121] Yuan F., Wang X., Zhao B., Xu X., Shi M., Leng B., Dong X., Lu C., Feng Z., Guo J. (2022). The genome of the recretohalophyte *Limonium bicolor* provides insights into salt gland development and salinity adaptation during terrestrial evolution. Mol. Plant.

[122] Velts Veltsos P., E Ridout K., A Toups M., González-Martínez S.C., Muyle A., Emery O., Rastas P., Hudzieczek V., Hobza R., Vyskot B. (2019). Early sex-chromosome evolution in the diploid dioecious plant *Mercurialis annua*. Genetics.

[123] Gómez J.M., González-Megías A., Narbona E., Navarro L., Perfectti F., Armas C. (2022). Phenotypic plasticity guides *Moricandia arvensis* divergence and convergence across the *Brassicaceae* floral morphospace. New Phytol..

[124] Li B., Peng L., Sun X.C., Huang W.J., Wang N., He Y.H., Shi X.B., Liu Y.R., Zhang P., Yang X.J. (2020). Organ-specific transcriptome sequencing and mining of genes involved in polyphyllin biosynthesis in *Paris polyphylla*. Ind. Crop. Prod..

[125] Safdari P., Höckerstedt L., Brosche M., Salojärvi J., Laine A.L. (2021). Genotype-Specific Expression and NLR Repertoire Contribute to Phenotypic Resistance Diversity in *Plantago lanceolata*. Front. Plant Sci..

[126] Kang J.N., Kim J.S., Lee S.M., Won S.Y., Seo M.S., Kwon S.-J. (2021). Analysis of phenotypic characteristics and sucrose metabolism in the roots of *Raphanus sativus* L. Front. Plant Sci..

[127] Yu R., Sun C., Zhong Y., Liu Y., Sanchez-Puerta M.V., Mower J.P., Zhou R. (2022). The minicircular and extremely heteroplasmic mitogenome of the holoparasitic plant *Rhopalocnemis phalloides*. Curr. Biol..

[128] Schelkunov M.I., Nuraliev M.S., Logacheva M.D. (2019). *Rhopalocnemis phalloides* has one of the most reduced and mutated plastid genomes known. PeerJ.

[129] Sandler G., Beaudry F.E., Barrett S.C., Wright S.I. (2018). The effects of haploid selection on Y chromosome evolution in two closely related dioecious plants. Evol. Lett..

[130] Rifkin J.L., Beaudry F.E.G., Humphries Z., Choudhury B.I., Barrett S.C.H., Wright S.I. (2021). Widespread Recombination Suppression Facilitates Plant Sex Chromosome Evolution. Mol. Biol. Evol..

[131] Su Y., Jiao M., Guan H., Zhao Y., Deji C., Chen G. (2023). Comparative transcriptome analysis of *Saposhnikovia divaricata* to reveal drought and rehydration adaption strategies. Mol. Biol. Rep..

[132] Angeloni F., Wagemaker C.A.M., Jetten M.S.M., Camp H.J.M.O.D., Janssen-Megens E.M., Francoijs K., Stunnenberg H.G., Ouborg N.J. (2011). De novo transcriptome characterization and development of genomic tools for Scabiosa columbaria L. using next-generation sequencing techniques. Mol. Ecol. Resour..

[133] Muyle A., Martin H., Zemp N., Mollion M., Gallina S., Tavares R., Silva A., Bataillon T., Widmer A., Glémin S. (2021). Dioecy is associated with high genetic diversity and adaptation rates in the plant genus *Silene*. Mol. Biol. Evol..

[134] Wang T.J., Ru D.F., Zhang D., Hu Q.J. (2019). Analyses of genome-scale variation reveal divergence of two *Sinalliaria* species (*Brassicaceae*) with continuous but limited gene flow. J. Syst. Evol..

[135] Liu J., Mei D., Li Y., Huang S., Hu Q. (2014). Deep RNA-Seq to unlock the gene bank of floral development in Sinapis arvensis. PLoS ONE.

[136] Ananda G.K.S., Norton S.L., Blomstedt C., Furtado A., Møller B.L., Gleadow R., Henry R.J. (2022). Transcript profiles of wild and domesticated sorghum under water-stressed conditions and the differential impact on dhurrin metabolism. Planta.

[137] Ranathunge C., Wheeler G.L., Chimahusky M.E., Kennedy M.M., Morrison J.I., Baldwin B.S., Perkins A.D., Welch M.E. (2018). Transcriptome profiles of sunflower reveal the potential role of microsatellites in gene expression divergence. Mol. Ecol..

[138] Guo Y., Zhang X., Wu H., Yu R., Zhang J., Zhu K.Y., Guo Y., Ma E. (2015). Identification and functional analysis of a cytochrome *P450* gene *CYP9AQ2* involved in deltamethrin detoxification from *Locusta migratoria*. Pestic. Biochem. Physiol..

[139] Bowsher A.W., Shetty P., Anacker B.L., Siefert A., Strauss S.Y., Friesen M.L. (2017). Transcriptomic responses to conspecific and congeneric competition in co-occurring *Trifolium*. J. Ecol..

[140] Wang C., Liu R., Liu Y., Hou W., Wang X., Miao Y., He Y., Ma Y., Li G., Wang D. (2021). Development and application of the Faba_bean_130K targeted next-generation sequencing SNP genotyping platform based on transcriptome sequencing. Theor. Appl. Genet..

[141] Kim B.Y., Park H.S., Kim S., Kim Y.D. (2017). Development of microsatellite markers for *Viscum coloratum* (*Santalaceae*) and their application to wild populations. Appl. Plant Sci..

