# Supplementary material for: Data Mining Sheds Light on a Novel Family of Plant-Associated Negative-Sense RNA Viruses Linked to Lispiviruses
Source: Viruses. 2026 Oct 8;18(10):1113. doi: 10.3390/v18101113 (PMC13709462; doi:10.3390/v18101113)
Supplement: Supplementary file 1 [file viruses-18-01113-s001.zip › Supplementary Table S1 dataset_counts.pdf]

Supplementary Table S1. Summary of dataset counts at each stage of sequence identification, filtering and classification. All counts reported elsewhere in the manuscript, in the figures and in Tables 1 and 2 refer back to this table.

Stage	Quantity	Value	Where reported
palmID search	Query protein	MSuV L (RdRp), GenBank MZ270532 / UWX11517.1	Section 2.1
palmID search	Palmprint extracted by palmID	113 aa (barcode u64591)	Contains motifs A–C (DFSKW, GWEGQRQKGW, QGDNQ)
palmID search	Related palmprints returned from palmDB	200	Identity range 38.9–100% to the MSuV palmprint
palmID search	SRA hits returned	1,347	Section 2.1
palmID search	Distinct SRA runs	1,202	Section 2.1
palmID search	Distinct sOTUs	185	Section 2.1
palmID search	Distinct labelled source organisms	198	Section 2.1
palmID search	Distinct BioProjects represented	255	Section 2.1
Retention	Criterion applied to each hit	BLASTP of palm-ID vs NCBI nr; best hit = MSuV or DpaLV	Section 2.1
Retention	BioProjects contributed by the archived single-query search	27 of 67	Section 2.1
Retention	BioProjects contributed by iterative searches and the TSA route	40 of 67	Section 2.1
Parallel route	TSA tBLASTn queries	MSuV N and L proteins vs Viridiplantae (taxid 33090)	Section 2.1
Filtering	Sequences retained with a complete coding region	87	Retention criterion; Table 1
Dataset	Coding-complete sequences analysed	87	Table 1; Figures 1–5
Dataset	Genome length range (nt)	11,193–13,620 (median 12,298)	Table 1
Dataset	Read coverage range (fold)	12–909 (median 64)	Table 1
Dataset	Independent NCBI BioProjects contributing sequences	67	Table 1; Section 3.16
Hosts	Distinct plant host species	75	Table 1
Hosts	Distinct plant host botanical families	25 (see note)	Table 1
Taxonomy	Proposed genera	15	Section 3.8; Alphamasuvirus–Omicronmasuvirus
Taxonomy	Proposed families	1	Masuviridae
Taxonomy	Virus–host associations analysed at genus level	89	Section 3.8
Logan screen	Query sequences used	89	87 new + MSuV + DpaLV; Section 2.14
Logan screen	Positive public RNA-seq libraries	1,536	Section 3.9
Logan screen	Distinct host plant species	134	Section 3.9
Logan screen	Distinct host plant families	11	Section 3.9
Logan screen	Distinct geographic locations	182	Section 3.9
Logan screen	Deposition years spanned	2015–2024 (9 years)	Section 3.9

Contamination control	BioProjects with a STAT profile available	66 of 67	Section 3.16; Supp. Table S3
Contamination control	Median Viridiplantae read fraction of source runs	72.8%	Supp. Fig. S8
Contamination control	Median Arthropoda read fraction of source runs	0.009%	Supp. Fig. S8
