# Supplementary material for: Data Mining Sheds Light on a Novel Family of Plant-Associated Negative-Sense RNA Viruses Linked to Lispiviruses
Source: Viruses. 2026 Oct 8;18(10):1113. doi: 10.3390/v18101113 (PMC13709462; doi:10.3390/v18101113)
Supplement: Supplementary file 1 [file viruses-18-01113-s001.zip › Supplementary Table S2 STAT.pdf]

Supplementary Table S2. SRA Taxonomy Analysis Tool (STAT) taxonomic composition of the source sequencing libraries. For each of the 66 independent BioProjects contributing sequences to Table 1 for which a precomputed STAT profile was available, a representative run is shown. The run shown represents its BioProject and is not necessarily the specific run from which the viral contig was assembled. The one remaining project, PRJNA763316, has no precomputed STAT profile for any of its runs. Percentages are expressed relative to the total number of taxonomically identified reads in that run. Plant : arthropod is the ratio of Viridiplantae-assigned to Arthropoda-assigned reads (computed with a pseudocount of 1 to accommodate runs with no arthropod-assigned reads).

Run	BioProject	Virus(es) in Table 1	Identified reads	Viridiplantae (%)	Metazoa (%)	Arthropoda (%)	Insecta (%)	Plant : arthropod
SRR7030595	PRJNA450088	Epipactis lispi-like virus 1; Epipactis lispi-like virus 2	8,794,996	99.23	0.037	0.0024	0.0014	41,757
SRR6467495	PRJNA309775	Brassica lispi-like virus 1	22,066,326	99.12	0.002	0.0000	0.0000	21,872,994
SRR3189868	PRJNA312975	Brassica lispi-like virus 2_rap	8,992,042	99.10	0.087	0.0018	0.0017	54,669
SRR3319778	PRJNA311722	Trifolium lispi-like virus 1	5,152,674	97.68	0.301	0.0062	0.0062	15,679
SRR5195396	PRJNA362874	Eruca lispi-like virus 1_ery	13,106,532	97.00	0.002	0.0000	0.0000	12,712,750
SRR8863732	PRJNA531360	Coriandrum lispi-like virus 1	8,108,670	96.42	0.702	0.0000	0.0000	7,818,706
SRR6453872	PRJNA428769	Brassica lispi-like virus 3_inc; Brassica lispi-like virus 3_ins; Brassica lispi-like virus 3_ole (+2 more)	8,626,709	96.12	0.003	0.0001	0.0000	1,036,488
SRR7289650	PRJNA475309	Eruca lispi-like virus 1_eru	6,046,664	95.97	0.029	0.0007	0.0007	138,166
SRR12366085	PRJNA650223	Raphanus lispi-like virus 1	11,956,354	95.84	0.045	0.0004	0.0003	238,731
SRR10271151	PRJNA577252	Centaurea lispi-like virus 1	8,960,321	94.22	0.774	0.0158	0.0158	5,949
SRR6061856	PRJNA408292	sunflower lispi-like virus 1	27,532,908	94.01	0.041	0.0116	0.0011	8,096
SRR11365145	PRJNA613937	Saposhnikovia lispi-like virus 1	4,289,240	92.76	0.406	0.0066	0.0015	14,109
SRR4473609	PRJNA350768	Viscum lispi-like virus 1	1,402,208	92.10	0.198	0.0866	0.0866	1,062
SRR18361965	PRJNA533007	Capsella lispi-like virus 1	29,510,239	91.65	0.010	0.0003	0.0001	275,990
SRR3995704	PRJNA335689	Ambrosia lispi-like virus 1	2,913,907	91.48	2.724	0.0341	0.0008	2,676
SRR13258417	PRJNA679419	Vicia lispi-like virus 1	7,459,262	91.01	3.738	2.8470	2.8449	32
SRR1867374	PRJNA277578	Cypripedium lispi-like virus 1	1,149,015	89.85	0.634	0.0715	0.0715	1,254
SRR7466533	PRJNA479379	Cypripedium lispi-like virus 2	1,683,360	89.27	1.203	0.0719	0.0716	1,240
SRR6853628	PRJNA438242	Heracleum lispi-like virus 1	2,694,757	87.87	1.683	0.0240	0.0232	3,660
SRR10201888	PRJNA574412	carrot lispi-like virus 1	34,534,485	86.09	0.021	0.0001	0.0001	571,728

SRR10215277	PRJNA575330	Arabidopsis lispi-like virus 1	12,587,375	86.05	0.035	0.0006	0.0001	133,720
SRR11902776	PRJNA636383	Plantago lispi-like virus 1	7,023,470	84.52	0.187	0.1703	0.1604	496
SRR1150769	PRJNA233448	Arnica lispi-like virus 1	414,722	84.27	0.694	0.1186	0.1167	709
SRR3757185	PRJNA328202	Cichorium lispi-like virus 1	98,650	83.68	1.382	0.0051	0.0051	13,759
SRR1066782	PRJNA232677	Sinapis lispi-like virus 1	25,649,313	83.63	0.042	0.0183	0.0183	4,560
SRR11547879	PRJNA625418	Cenchrus lispi-like virus 1	29,745,363	83.25	0.039	0.0074	0.0068	11,307
SRR14792181	PRJNA736757	Sorghum lispi-like virus 1	55,815,498	81.13	0.001	0.0000	0.0000	5,660,688
SRR7212185	PRJNA472685	Coriandrum lispi-like virus 2	2,830,398	79.89	1.320	1.1250	1.1250	71
SRR6441715	PRJNA428441	Sinalliaria lispi-like virus 1	15,455,681	79.01	0.028	0.0001	0.0001	555,036
SRR15384544	PRJNA752802	Limonium lispi-like virus 1	1,586,545	78.49	0.899	0.0037	0.0024	21,105
SRR6655828	PRJNA431031	Orychophragmus lispi-like virus 1	12,314,926	78.17	0.027	0.0000	0.0000	9,626,354
SRR7182974	PRJNA472058	Rumex lispi-like virus 1	686,652	74.85	2.704	0.1692	0.1657	442
SRR11003472	PRJNA604514	Moricandia lispi-like virus 1	17,219,970	74.27	0.440	0.0037	0.0029	20,140
SRR9329508	PRJNA549345	Brassica lispi-like virus 2_nap	18,069,755	71.33	0.063	0.0519	0.0518	1,375
SRR16550906	PRJNA773629	Cannabis lispi-like virus 1	96,800,941	69.25	0.042	0.0006	0.0001	110,251
SRR15728919	PRJNA760819	Heliosperma lispi-like virus 1	3,787,353	68.69	0.243	0.0017	0.0010	40,646
SRR17026842	PRJNA783190	Paris lispi-like virus 1	4,250,552	67.57	3.130	0.0445	0.0150	1,516
SRR6325660	PRJNA419787	Atractylodes lispi-like virus 1	19,616,665	62.82	0.246	0.0642	0.0599	978
SRR331931	PRJNA80113	Scabiosa lispi-like virus 1	4,906,330	61.49	5.826	0.0012	0.0012	49,454
SRR10829330	PRJNA598927	Cistanche lispi-like virus 1; Cistanche lispi-like virus 2	5,440,331	60.52	0.523	0.0605	0.0307	999
SRR10664371	PRJNA542932	Allium lispi-like virus 1; Allium lispi-like virus 2	3,865,368	59.15	0.924	0.0238	0.0198	2,483
SRR14800310	PRJNA737177	Rhopalocnemis lispi-like virus 1; Rhopalocnemis lispi-like virus 2; Rhopalocnemis lispi-like virus 3	6,854,653	57.18	0.925	0.0445	0.0199	1,285
SRR13623198	PRJNA698922	Rumex lispi-like virus 2	1,349,546	56.50	3.380	1.5524	1.5499	36
SRR11818586	PRJNA633672	Allium lispi-like virus 3	3,325,607	55.27	0.945	0.0026	0.0022	20,886
DRR031853	PRJDB3033	Monotropastrum lispi-like virus 1	2,437,662	54.61	0.517	0.0009	0.0000	60,506
SRR5460706	PRJNA38309	Suaeda lispi-	11,687,495	53.61	1.465	0.1342	0.0123	400

	2	like virus 1						
SRR10189295	PRJNA574279	Ophrys lispi-like virus 1; Ophrys lispi-like virus 2	2,637,330	52.45	0.840	0.0048	0.0031	10,892
SRR10013331	PRJNA561399	Cuscuta lispi-like virus 1	4,337,627	51.91	0.693	0.0665	0.0023	780
SRR14038219	PRJNA662860	Coptis lispi-like virus 1	2,087,116	48.88	0.705	0.0233	0.0221	2,090
SRR8175630	PRJNA504609	Gymnadenia lispi-like virus 1	49,602	48.05	1.536	0.0101	0.0101	3,973
SRR11119228	PRJNA607615	Erysimum lispi-like virus 1	61,766,752	46.96	0.119	0.0013	0.0009	37,232
SRR2807657	PRJNA203407	Dionaea lispi-like virus 1	118,704,755	46.54	0.226	0.0709	0.0465	656
SRR9202867	PRJNA369310	Mercurialis lispi-like virus 1	8,451	45.25	27.015	1.1715	1.1715	38
ERR4643784	PRJEB39526	Silene lispi-like virus 1_lat; Silene lispi-like virus 1_heu; Silene lispi-like virus 2	1,214,724	44.04	0.963	0.0000	0.0000	534,927
SRR5237276	PRJNA371565	Ageratum lispi-like virus 1; Ageratum lispi-like virus 2	24,713,919	40.38	0.551	0.0757	0.0277	534
SRR12619942	PRJNA661963	Caladenia lispi-like virus 1_att; Caladenia lispi-like virus 2_act; Caladenia lispi-like virus 2_cre	11,396,176	39.57	0.151	0.0041	0.0031	9,738
SRR6127586	PRJNA412930	Ixiolirion lispiu-like virus 1	3,415,899	38.23	1.805	0.0362	0.0107	1,054
SRR2175825	PRJNA293262	Erigeron lispi-like virus 1	11,933,034	32.35	0.298	0.1168	0.0984	277
SRR17665593	PRJNA317244	Dactylorhiza lispi-like virus 1; Dactylorhiza lispi-like virus 2; Dactylorhiza lispi-like virus 3	107,991,110	32.30	0.163	0.0012	0.0007	26,052
SRR5487523	PRJNA384875	Caladenia lispi-like virus 1_pli	3,354,741	25.31	0.715	0.0319	0.0317	794
SRR13390672	PRJNA690069	Astragalus lispi-like virus 1	4,777,412	21.05	1.368	0.0121	0.0105	1,737
SRR5558418	PRJNA386610	Lactuca lispi-like virus 1	9,572,613	16.44	0.086	0.0033	0.0009	4,949
SRR5985187	PRJNA399494	Tanacetum lispi-like virus 1	4,196,902	12.00	0.578	0.0969	0.0969	124
SRR12771435	PRJNA495456	Rhopalocnemis lispi-like virus 4; Rhopalocnemis lispi-like virus 5; Rhopalocnemis lispi-like virus 6 (+1 more)	11,499,793	8.15	15.546	1.1512	0.8217	7
SRR3085405	PRJNA307530	Arceuthobium lispi-like virus 1	211,610	7.62	0.595	0.0014	0.0009	4,031

ERR348743 0	PRJEB21674	Phacelia lispi-like virus 1	642,891	1.54	0.964	0.0012	0.0005	1,102

Summary. n = 66 runs from 66 independent BioProjects. Viridiplantae: median 72.8%, range 1.5–99.2%. Arthropoda: median 0.009%, mean 0.146%, maximum 2.85%. Arthropoda < 0.1% in 56/66 runs, < 0.01% in 33/66 runs, and absent in 5 runs.
